# Chemokines protect vascular smooth muscle cells from cell death induced by cyclic mechanical stretch

**DOI:** 10.1038/s41598-017-15867-8

**Published:** 2017-11-23

**Authors:** Jing Zhao, Yuhei Nishimura, Akihiko Kimura, Kentaro Ozawa, Toshikazu Kondo, Toshio Tanaka, Masanori Yoshizumi

**Affiliations:** 1 Department of Pharmacology, Nara Medical University School of Medicine, Nara, Japan; 2 Department of Molecular and Cellular Pharmacology, Pharmacogenomics and Pharmacoinformatics, Tsu, Mie Japan; 3 Mie University Medical Zebrafish Research Center, Tsu, Mie Japan; 4 Department of Bioinformatics, Mie University Life Science Research Center, Tsu, Mie Japan; 5 Department of Omics Medicine, Mie University Industrial Technology Innovation Institute, Tsu, Mie Japan; 6 Department of Systems Pharmacology, Mie University Graduate School of Medicine, Tsu, Mie Japan; 7 Department of Forensic Medicine, Wakayama Medical University, Wakayama, Japan; 8 Department of Psychiatry, Osaka University Graduate School of Medicine, Osaka, Japan

## Abstract

The pulsatile nature of blood flow exposes vascular smooth muscle cells (VSMCs) in the vessel wall to cyclic mechanical stretch (CMS), which evokes VSMC proliferation, cell death, phenotypic switching, and migration, leading to vascular remodeling. These responses have been observed in many cardiovascular diseases; however, the underlying mechanisms remain unclear. We have revealed that CMS of rat aortic smooth muscle cells (RASMCs) causes JNK- and p38-dependent cell death and that a calcium channel blocker and angiotensin II receptor antagonist decreased the phosphorylation of JNK and p38 and subsequently decreased cell death by CMS. In the present study, we showed that the expression of *Cxcl1* and *Cx3cl1* was induced by CMS in a JNK-dependent manner. The expression of *Cxcl1* was also induced in VSMCs by hypertension produced by abdominal aortic constriction (AAC). In addition, antagonists against the receptors for CXCL1 and CX3CL1 increased cell death, indicating that CXCL1 and CX3CL1 protect RASMCs from CMS-induced cell death. We also revealed that STAT1 is activated in RASMCs subjected to CMS. Taken together, these results indicate that CMS of VSMCs induces inflammation-related gene expression, including that of CXCL1 and CX3CL1, which may play important roles in the stress response against CMS caused by hypertension.

## Introduction

Hypertension is the most important preventable risk factor for cardiovascular diseases, including ischemic heart disease, stroke, and aortic aneurysms^1^. Hypertension causes vascular remodeling, which contributes to the development of cardiovascular diseases. Proliferation, cell death, inflammation, and fibrosis are all mechanisms that have been suggested to contribute to arterial remodeling in hypertension^2^. Cell death has been reported to occur in vascular smooth muscle cells (VSMCs) in the blood vessels of animal models of hypertension^3,4^; however, there have been some reports that cell death is reduced in VSMCs in the small arteries of young spontaneously hypertensive rats (SHR)^5^, suggesting that hypertension has a two-sided effect on cell death. Many factors have been reported to be involved in VSMC death induced by hypertension, including reactive oxygen species^6^, NO^7^, angiotensin II^8^, and endothelin^9^; however, the pathways that cause cell death by hypertension remain unclear.

The pulsatile nature of blood pressure creates hemodynamic stimuli in the form of cyclic mechanical stretch (CMS) that acts on the constituents of the blood vessel walls, and VSMCs are primarily subjected to CMS resulting from pulsatile blood pressure^2^. We have revealed that CMS causes cell death in rat aortic smooth muscle cells (RASMCs) in JNK- and p38-dependent manners^10^ and that a dihydropyridine calcium channel blocker, Azelnidipine^10^, and an angiotensin II receptor antagonist, Olmesartan^11^, inhibit the phosphorylation of JNK and p38 and protect RASMCs from cell death caused by CMS. These findings suggest that hypertension may be a dominant cause of cell death in VSMCs in a MAP kinase-dependent manner and that Azelnidipine and Olmesartan may be used as pharmacotherapeutic agents for the prevention of vascular dysfunction, including aortic dissection and atherosclerosis, independent of their blood pressure-lowering effect.

In this study, to investigate the mechanisms involved in cell death in VSMCs caused by CMS, we employed RASMCs to compare to our previous study and compared gene expression between control and CMS-subjected RASMCs; we also identified the expression of two chemokines, *Cxcl1* and *Cx3cl1*, that were induced by CMS in a JNK-dependent manner. To clarify the roles of CXCL1 and CX3CL1 in cell death induced by CMS, we evaluated the secretion of the chemokines in supernatant and then evaluated the effect of the chemokines on cell death. Furthermore, we investigated the effects of CXCL1 and CX3CL1 on CMS-induced VSMC death using antagonists against the receptors for CXCL1 and CX3CL1.

## Results

### The transcriptome analysis revealed the potential association between MAP kinase and cell death

To compare the gene expression of primary RASMCs subjected to CMS with the gene expression under normal conditions using Agilent arrays, RASMCs were prepared, cultured, and subjected to CMS (60 cycles/min, 15% elongation) for 4 hours as described in the Materials and Methods section. The 4-hour duration was selected as the time point at which cell death is initiated in RASMCs based on our previous studies^10^. Total RNA was purified and analyzed with microarrays as described in the Materials and Methods section.

**Figure 1:**
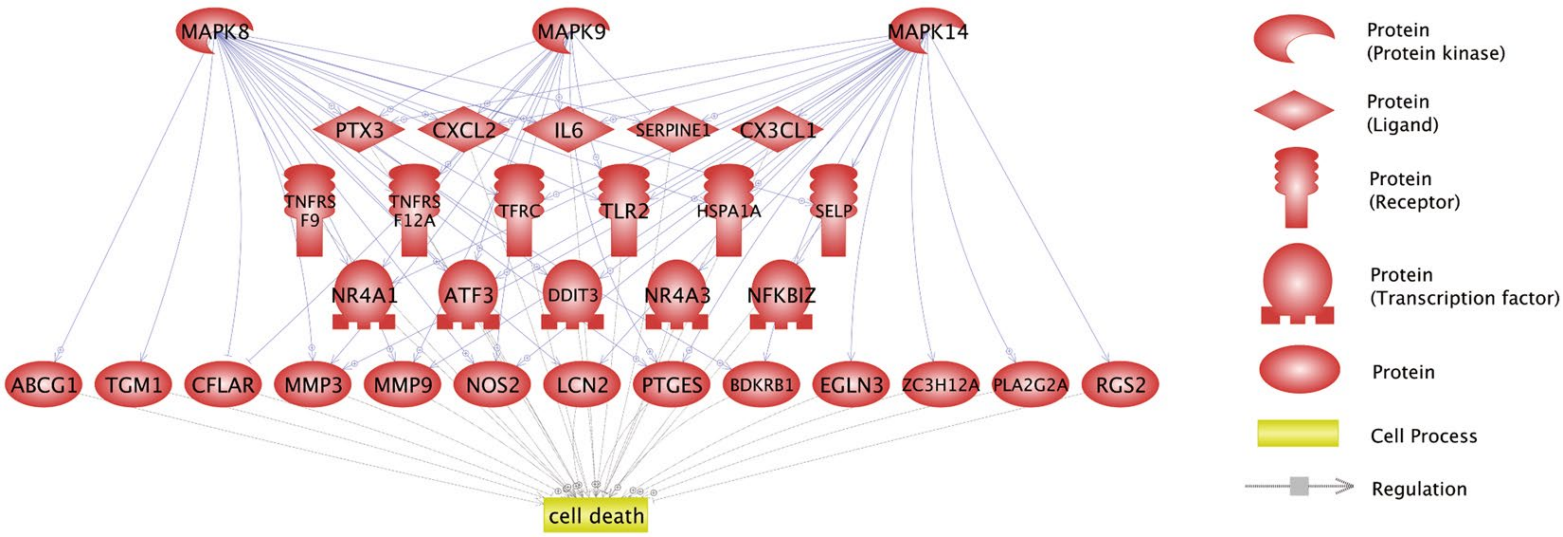
Molecular networks associated with JNK/p38 MAP kinase and cell death. The 91 genes dysregulated in RASMCs treated with cyclic mechanical stretch were subjected to bioinformatics analyses to identify genes that are related to both JNK and p38 MAP kinases and cell death. The regulations among the MAP kinases, dysregulated genes, and cell death are shown in the network.

We previously demonstrated that JNK (MAPK8 and MAPK9) and p38 MAP kinase (MAPK14) are involved in the death of RASMCs induced by CMS^10^. To identify genes related to the death of RASMCs regulated by JNK, we analyzed data from the microarray. As shown in Table S1, 91 differentially expressed genes (DEGs) were identified. The 91 DEGs were then subjected to Pathway Studio^12^ to identify the potential expression regulators and the cell processes significantly related to the 91 genes. As shown in Table S2–1, 71 regulators were identified, including MAPK14 (*p* = 1.04 × 10^−17^), MAPK8 (*p* = 3.24 × 10^−15^), and MAPK9 (*p* = 3.86 × 10^−14^). As shown in Table S2–2, 28 cell processes were identified, including cell death (*p* = 1.71 × 10^−11^). These analyses revealed that 29 of the 91 DEGs were potentially regulated by MAPK8, MAPK9, and/or MAPK14 and were related to cell death. The molecular networks are shown in Fig. 1. To identify the KEGG pathway significantly related to the 29 DEGS, we performed a bioinformatics analysis using WebGestalt^13^. As shown in Table S3, the “cytokine-cytokine receptor interaction” was significantly (*p* = 9.32 × 10^−6^) enriched in the 29 DEGs.

### CMS changes the expression of chemokine genes in RASMCs

**Figure 2:**
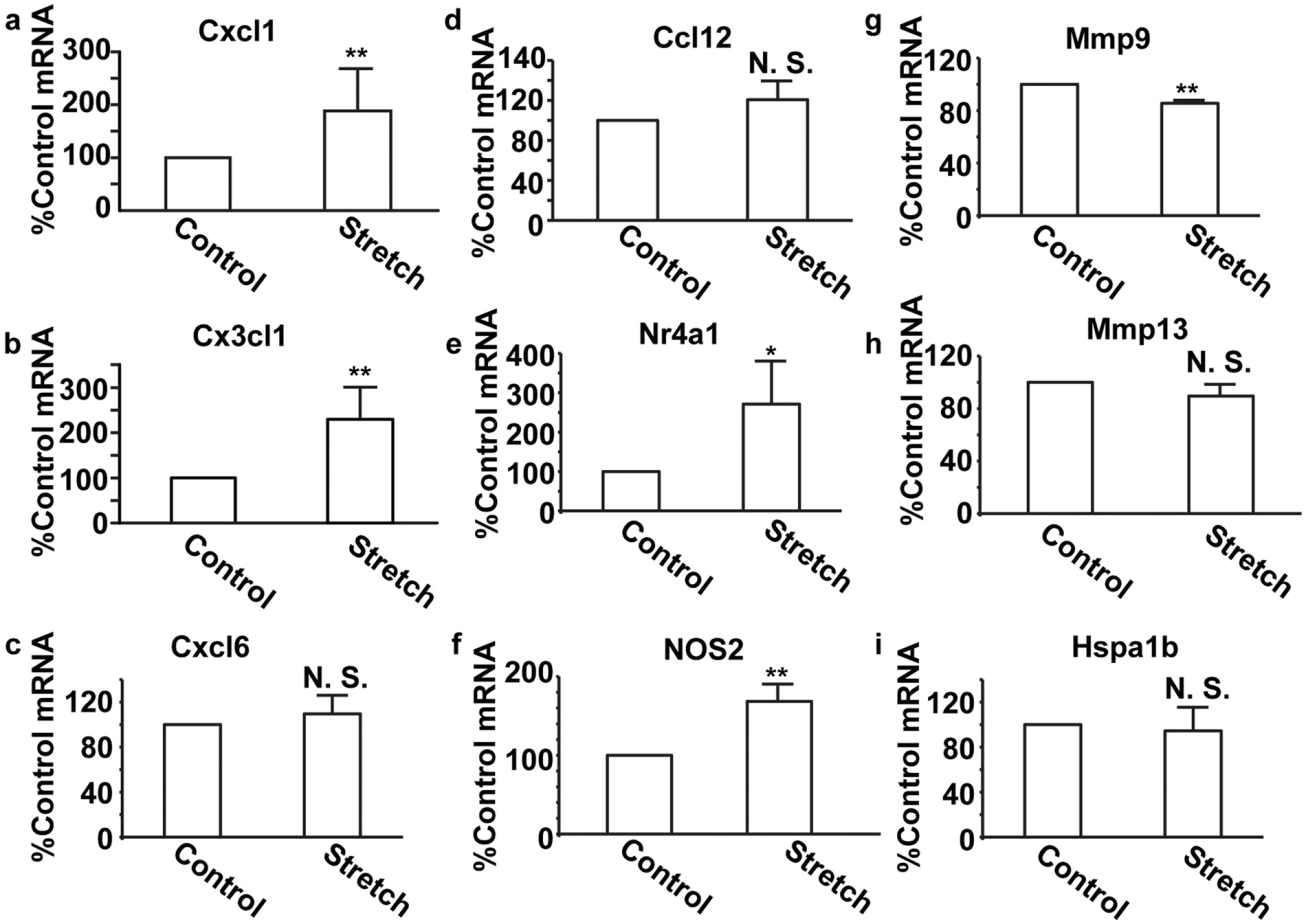
Expression of transcripts of candidate genes in RASMCs subjected to CMS. RASMCs were subjected to CMS for four hours, and expressions of transcripts of *Cxcl1* (**a**), *Cx3cl1* (**b**), *Cxcl6* (**c**), *Ccl12* (**d**), *Nr4a1* (**e**), *NOS2* (**f**), *Mmp9* (**g**), *Mmp13* (**h**), and *Hspa1b* (**i**) were evaluated using the real-time RT-PCR method. The quantity of the transcripts is expressed as a percentage of the control, normalized with respect to GAPDH. Data are means ± SE (n = 4); *p < 0.05 and **p < 0.01 versus control, and N.S. indicates no significant difference.

The results obtained from the array and transcriptome analysis showed that the expression of some genes involved in inflammation, including chemokines, may be increased; therefore, real-time polymerase chain reaction (PCR) assays were performed to confirm the chemokine transcripts that were induced by CMS. Transcripts of *Cxcl1* and *Cx3cl1* were significantly increased in RASMCs subjected to CMS (Fig. 2a and b), whereas those of Cxcl6 and Ccl12 showed no significant changes (Fig. 2c and d). Transcripts of *Nr4a1* and *Nos2* were significantly increased in RASMCs subjected to CMS (Fig. 2e and f), which is in accordance with previous reports^14,15^, suggesting that the array experiments should have worked well. In addition, transcripts of *Mmp9* and *Mmp13*, which have been reported to be induced by CMS^16,17^, were not increased (Fig. 2g and h). The difference might stem from a different incubation time of CMS because the induction of some metalloproteases, including Mmp9, is regulated in a time-dependent manner^18^. Heat shock proteins were reported to be induced by CMS in coronary artery fibroblasts^19^; however, *Hspa1b* expression was not significantly different between control and CMS-subjected RASMCs (Fig. 2i), suggesting that the mechanisms associated with transcript induction by CMS may differ between cell types.

### CMS changes the transcripts and the products of *Cxcl1* and *Cx3cl1* in a JNK-dependent manner

**Figure 3:**
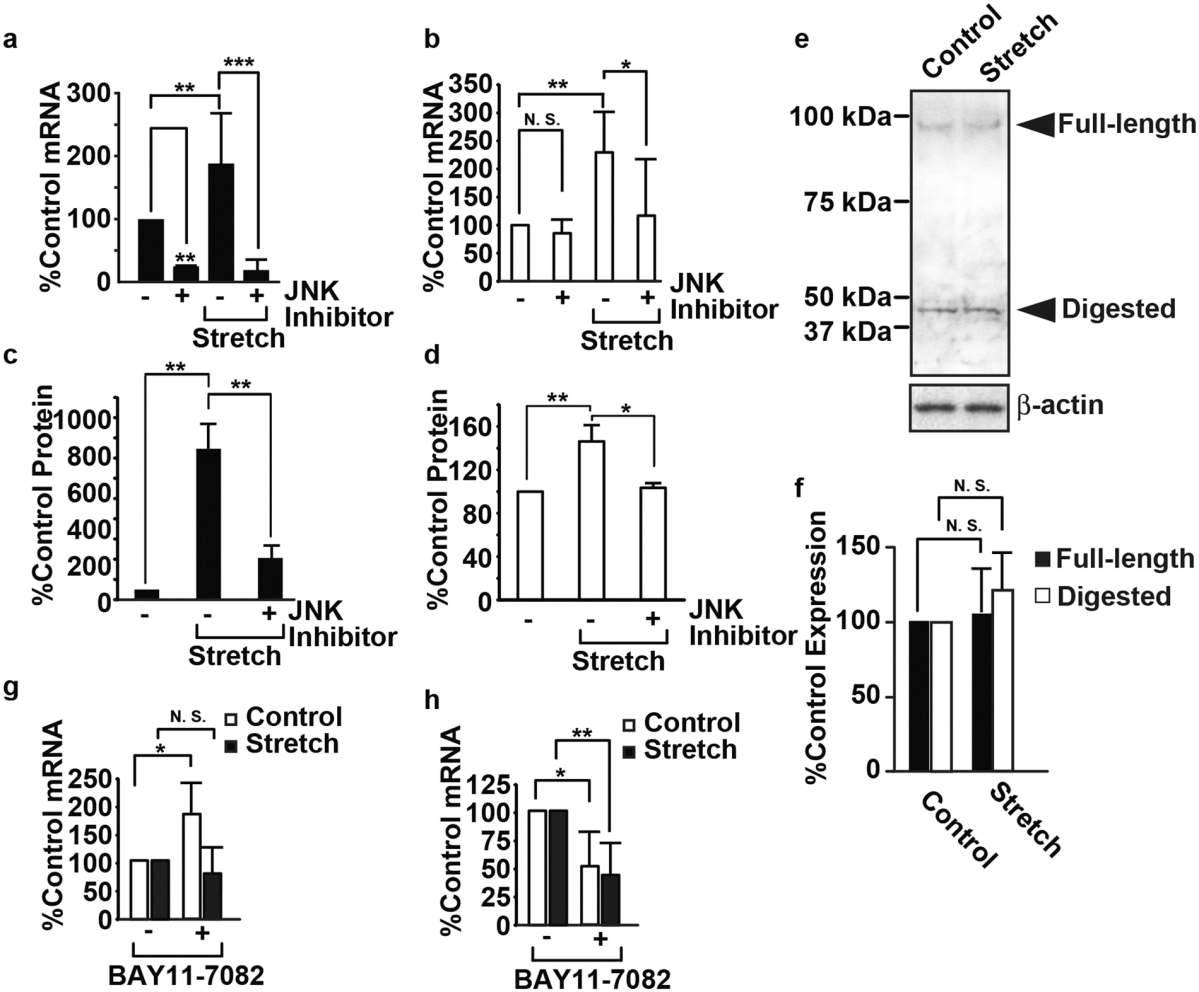
Induction of CXCL1 and CX3CL1 in RASMCs subjected to CMS. RASMCs were incubated with SP600125 (20 μM) for 20 min and then subjected to CMS for four hours. Cells were harvested and analyzed by real-time RT-PCR with specific primers for *Cxcl1* (**a**) or *Cx3cl1* (**b**) or by immunoblotting using the anti-CX3CL1 antibody (**e**), and media were analyzed using ELISA for CXCL1 (**c**) or CX3CL1 (**d**). (**f**) The quantity of CX3CL1 expressed in RASMCs, as measured by scanning densitometry, is expressed as a percentage of the control, normalized with respect to beta-actin. Data are means ± SE (n = 3), and N.S. indicates no significant difference. RASMCs were incubated with BAY 11-7082 (5 µM) for 20 min and then subjected to CMS for four hours. Cells were harvested and analyzed by real-time RT-PCR with specific primers for *Cxcl1* (**g**) or *Cx3cl1* (**h**). The uncropped pictures of immuoblotting are shown in Supplemental Fig. S4 (**a**,**b**).

Our previous reports have shown that the viability of RASMCs subjected to CMS decreased in a JNK-dependent manner^10^. Therefore, we evaluated the transcripts and products of *Cxcl1* and *Cx3cl1* using a JNK inhibitor, SP600125. The transcripts of *Cxcl1* with and without CMS were decreased by SP600125 (Fig. 3a), indicating that the expression of *Cxcl1* is transcriptionally dominated by JNK. The transcripts of *Cx3cl1* associated with CMS were decreased by SP600125, whereas there was no significant change in the absence of CMS (Fig. 3b). SB203580, a p38 inhibitor, did not significantly change the expression of *Cxcl1* and *Cx3cl1* (Supplemental Fig. S2). This result suggests that the induction of *Cx3cl1* by CMS may be controlled by JNK, and basal expression may be controlled by other pathways. CXCL1 and CX3CL1 secreted from RASMCs were induced by CMS in a JNK-dependent manner (Fig. 3c and d), whereas the expression of CX3CL1 in RASMCs showed no significant change with or without CMS (Fig. 3e and f). We also evaluated the transcripts of *Cxcl1* and *Cx3cl1* using BAY11-7082, a selective inhibitor of the phosphorylation of I-kappaB^20^. The transcripts of *Cxcl1* without CMS were increased by BAY11-7082 (Fig. 3g), whereas the transcripts of *Cxcl1* with CMS were unchanged, indicating that the induction of *Cxcl1* by CMS is independent of NF-kappaB. The transcripts of *Cx3cl1* with and without CMS were decreased by BAY11-7082 (Fig. 3h), indicating that the consistent expression of *Cx3cl1* was regulated in a NF-kappaB-dependent manner. We have reported that a calcium blocker and an angiotensin II receptor antagonist decreased cell death in RASMCs subjected to CMS; hence, we evaluated the transcripts of *Cxcl1* and *Cx3cl1* in RASMCs subjected to CMS with Azelnidipin and Olmesartan; neither drug significantly changed the expression of the chemokines, possibly because of their partial effect on the phosphorylation of JNK (Supplemental Fig. S6)^10,11^.

### Hypertension induces the expression of CXCL1 in vascular smooth muscle cells

**Figure 4:**
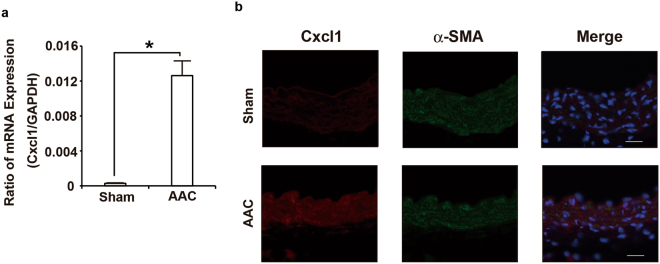
AAC-induced CXCL1 expression in the aorta. (**a**) Real-time RT-PCR analysis of *Cxcl1* gene expression in the aorta of sham and AAC mice 6 h post-operation. (**b**) Double-color immunofluorescence images of CXCL1 (red) and a-SMA (green) in the aortas of sham and AAC mice 6 h post-operation. Representative results from four individual animals in each group are shown. Original magnification, ×400 (scale: 25 mm). All values represent means ± SEM (n4–6). *p < 0.05, AAC versus sham.

To investigate whether hypertension induces the expression of the chemokines in VSMCs, the abdominal aortic constriction (AAC) model, which induces sustained elevation of mean arterial pressure without the use of a vasopressor^21^, was employed. The levels of *Cxcl1* transcripts in arteries subjected to AAC for six hours were increased compared to those in arteries subjected to the sham-operated treatment (Fig. 4a), whereas the transcripts of *Cx3cl1* showed no significant changes (Supplemental Fig. S1). To confirm that CXCL1 was expressed in SMCs, an immunofluorescence analysis using antibodies against CXCL1 and alpha-SMA, which is a maker of VSMCs, was performed. The immunofluorescence analysis demonstrated that CXCL1 signals were significantly increased in the arteries subjected to AAC compared to the sham-operated arteries and overlapped with alpha-SMA signals (Fig. 4b). Consistent with our data on RASMCs subjected to CMS, these data indicated that the expression of CXCL1 in SMCs is induced early in the course of hypertension.

### Effects of CXCL1 and CX3CL1 on cell viability in RASMCs

**Figure 5:**
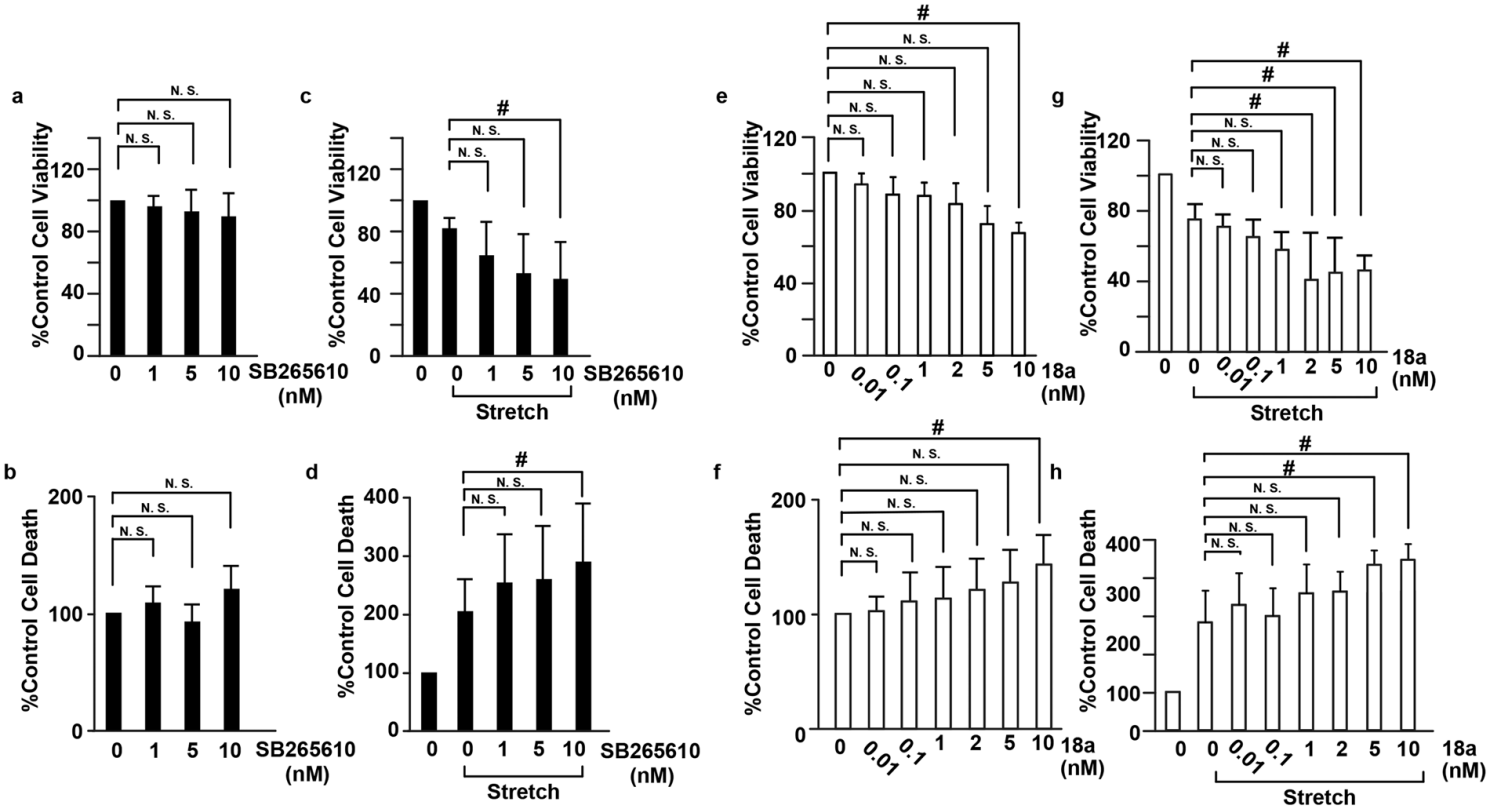
Inhibition of CXCL1 and CX3CL1 accelerated cell death induced by CMS. RASMCs were incubated with SB265610 (**a**–**d**) or 18a (**e**–**h**) at the indicated concentrations and then incubated under normal conditions (**a**,**b**,**e**,**f**) or subjected to CMS for four hours (**c**,**d**,**g**,**h**). After CMS, the cells were incubated for 24 h, and cell viability and cell death were evaluated by the MTT assay (**a**,**c**,**e**,**g**) and the release of LDH (**b**,**d**,**f**,**h**), respectively. Cell viability and cell death are expressed as a percentage of the control. Data are means ± SE (n = 6); *p < 0.05 versus control, and N.S. indicates no significant difference; ANOVA, Tukey’s HSD test.

The receptors for CXCL1 (CXCR2) and CX3CL1 (CX3CR1) are expressed in VSMCs^22^ (Supplemental Fig. S2), and we hypothesized that CXCL1 and CX3CL1 secreted from RASMCs play roles in cell death by binding to the receptors on RASMCs. To evaluate the role of CXCL1 and CX3CL1 on the viability of RASMCs subjected to CMS, RASMCs were incubated with antagonists against CXCR2 and CX3CR1 and then subjected to CMS. Although SB 265610, a CXCR2 antagonist, did not significantly change the viability or cell death (reflected by LDH released into the medium) of RASMCs not subjected to CMS (Fig. 5a and b), it decreased the viability and increased the death of RASMCs that were subjected to CMS (Fig. 5c and d). The CX3CR1 antagonist 18a showed similar tendencies to SB 265610 with respect to the viability of RASMCs with and without CMS, and a higher concentration of the CX3CR1 antagonist 18a decreased the viability and increased the death of RASMCs without CMS, consistent with the result that CX3CR1 was expressed in RASMCs without CMS (Fig. 3c and d). In addition, recombinant CXCL1 yielded no significant change in the proliferation of RASMCs (Supplemental Fig. S3). These data indicated that CXCL1 and CX3CL1 protect RASMCs from CMS-induced cell death in an autocrine manner.

### Phosphorylation of Stat1 is induced by cyclic mechanical stretch in RASMCs

**Figure 6:**
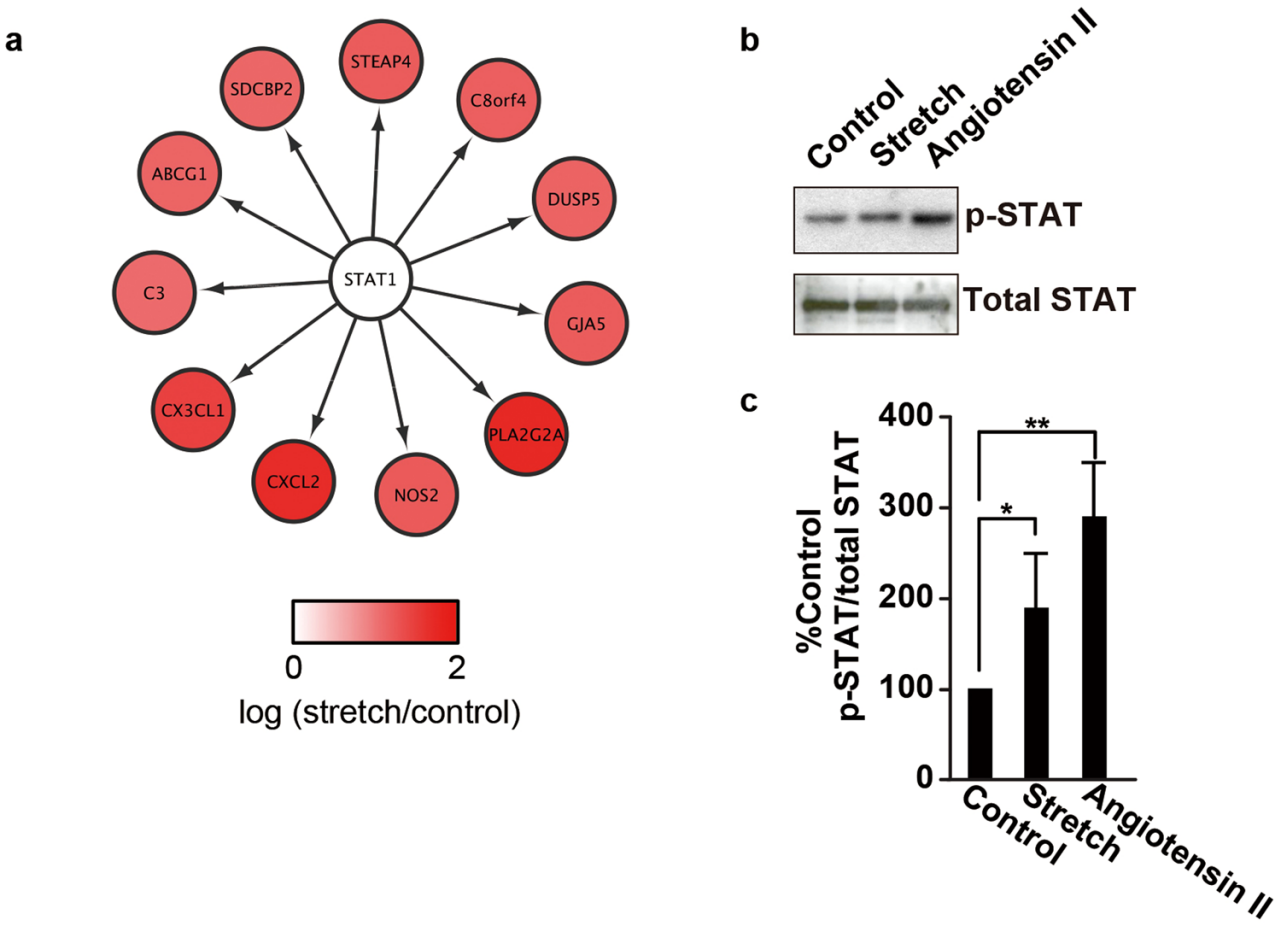
Phosphorylation of Stat1 is induced by mechanical stretch in RASMCs. (**a**) The transcriptome analysis identified STAT1 as the key transcription factor regulating the expression of genes induced by CMS in RASMCs. (**b**) RASMCs were incubated under normal conditions, subjected to CMS for four hours or to 10 nM angiotensin II for 5 min (**e**–**h**), and then harvested. Cell lysates were analyzed by immunoblotting using anti-phosphorylated (upper panel) and total STAT (lower panel) antibodies. (**c**) The quantity of phosphorylated STAT is expressed as a percentage of the control, normalized with respect to total STAT. Data are means ± SE (n = 3); *p < 0.05 and **p < 0.01 versus the control. The uncropped pictures of immuoblotting are shown in Supplemental Fig. S4 (**c**,**d**).

To investigate the mechanisms by which cell death is regulated by CMS in RASMCs, we performed a bioinformatics analysis using iRegulon^23^ with the same data as that used to generate Fig. 1. As shown in Table S4 and Fig. 6a, the analysis revealed STAT1 as a key transcription factor potentially regulating the 11 DEGs, including CXCL2 (the human homolog of rat *Cxcl1*) and CX3CL1. Immunoblotting using anti-phosphorylated STAT antibodies confirmed that STAT was phosphorylated in RASMCs subjected to CMS (Fig. 6b and c). Furthermore, we evaluated the phosphorylation of STAT1 in RASMCs with SP600125 (20 µM) or SB203580 (20 µM); neither changed the phosphorylation of STAT significantly, indicating that the phosphorylation of STAT1 is upstream of the phosphorylation of JNK (Supplemental Fig. S7).

## Discussion

Some secreted protein molecules, including monocyte chemoattractant protein-1 (MCP-1), stromal cell-derived factor-1 α (SDF-1α) and C-X-C chemokine receptor type 4 (CXCR4), are up-regulated by CMS^24,25^, which might play important roles in the pathogenic mechanism of vascular diseases through enhancing monocyte recruitment and smooth muscle cell migration. In this study, we showed that CMS induces two chemokines, CXCL1 and CX3CL1, in a JNK-dependent manner and that CXCL1 is induced in hypertension by AAC. Experiments using inhibitors against receptors of the chemokines CXCL1 and CX3CL1 protected SMCs from cell death by CMS, which should inhibit the pathogenesis of vascular diseases. We also revealed that STAT is activated in RASMCs subjected to CMS by bioinformatics analyses and confirmed this result by immunoblotting.

The pulsatile nature of blood flow exposes the VSMCs in blood vessels to CMS^2^. Under physiological conditions, the aorta undergoes an approximately 10% circumferential strain during systole^26^, and this number increases in conditions of hypertension, which should alter the phenotype of VSMCs. To investigate the effects of CMS on VSMCs and their associated mechanisms, systems to culture VSMCs and subject them to CMS *in vitro* have been widely used. In this study, CXCL1 increased more obviously than CX3CL1 in RASMCs subjected to CMS (Fig. 3b and d), whereas *Cxcl1*, but not *Cx3cl1*, was increased in hypertension *in vivo* (Fig. 4 and Supplemental Fig. S1), suggesting that CMS *in vitro* mimics hypertension *in vivo* with regard to the expression of CXCL1 and CX3CL1.

In this study, we revealed that CXCL1 and CX3CL1 protect VSMCs from cell death induced by CMS in an autocrine manner (Fig. 5), which should lead to suppressed vascular remodeling; however, CXCL1 and CX3CL1 have been reported to promote atherosclerosis^27^. CXCL1, secreted from endothelial cells, enhances arterial inflammation by its neutrophil chemoattractant activity^28^, and CX3CL1 inhibits the cell death of monocytes and foam cells in atheromatous plaques^29^, both of which promote vascular remodeling and subsequent atherogenesis. Taking into account the reports that polymorphisms in the CX3CL1 receptor CX3CR1 are a genetic risk factor for atherosclerosis^30^ and that the deletion of CXCL1^31^, CX3CL1^32^, and CX3CR1^33^ inhibits atherogenesis, CXCL1 and CX3CL1 should, overall, promote vascular remodeling by enhancing inflammation.

A bioinformatics analysis using iRegulon^23^ identified STAT1 as a key modulator of the expression of inflammation-related genes, including *Cxcl2* and *Cx3cl1*, and immunoblotting confirmed that STAT1 is phosphorylated in RASMCs subjected to CMS. Together with the finding that *Cxcl1* and *Cx3cl1* expression was induced in a JNK-dependent manner, these results indicate that phosphorylation of STAT1 may be regulated by JNK, or vice versa. To clarify the role of STAT1 on the induction of *Cxcl1* and *Cx3cl1*, we evaluated phosphorylation of STAT1 in RASMCs subjected to CMS with a JNK-inhibitor and a JNK-inhibitor did not change phosphorylation of STAT1, suggesting that STAT1 activation may be upstream JNK activation in RASMCs subjected to CMS, leading to induction of Cxcl1 and Cx3cl1.

Based on our findings, we suggest that the induction of chemokines exerts a protective effect against the death of VSMCs, which can, in turn, inhibit vascular remodeling. Our findings support the idea that the protection of VSMCs by chemokines may provide a novel therapeutic modality. However, because the chronic use of chemokines could exacerbate vascular remodeling by promoting inflammation^27–33^, the acute use of chemokines may protect VSMCs from cell death caused by an acute rise in blood pressure, which can lead to aortic dissection.

## Materials and Methods

### Approval

All methods were performed in accordance with relevant guidelines and regulations. All experimental protocols were approved by the institutional committees.

### Materials

Collagen I was purchased from Nippon Meat Packers, Inc. (Osaka, Japan). SB 265610 and CX3CR1 antagonist 18a were purchased from Axon Medchem (Reston, VA). ELISA kits for rat CXCL1 and CX3CL1 were purchased from RayBiotech, Inc. (Norcross, GA).

### Cell culture

The study design was approved by an ethics review board of guidelines for the use of laboratory animals of Nara Medical University (#11011). RASMCs were isolated from the thoracic aortas of 8-week-old male Sprague-Dawley rats as previously described^10^. Cells were grown in Dulbecco’s modified Eagle’s medium (DMEM, Nacalai, Japan) supplemented with 10% fetal bovine serum (FBS, HyClone, Logan, UT), penicillin (100 U/mL, Nacalai, Japan), and streptomycin (100 mg/mL, Nacalai, Japan) at 37 °C under 5% CO_2_ in a humidified incubator. RASMCs were used for experiments between the third and sixth passages. All chemical compounds were dissolved in dimethyl sulfoxide (DMSO) at a final concentration of less than 1% unless indicated otherwise. The tissue culture method was approved by the committee of Nara Medical University.

### Cyclic mechanical stretch (CMS)

The cells were cultured in collagen I-coated silicon chambers (STREX Inc., Osaka, Japan). When the cell confluency in culture was 70–80%, the medium was replaced with unsupplemented DMEM. The cells were further cultured for 24 h and then subjected to CMS (60 cycles/min, 15% elongation) for four hours using the computer-controlled mechanical strain unit (STREX Inc., Osaka, Japan). After cyclic stretch, the medium was replaced with DMEM containing 0.1% FBS. The CMS method was approved by the committee of Nara Medical University.

### Gene expression analysis

Total RNA was extracted with RNeasy columns (Qiagen, Valencia, CA) according to the manufacturer’s protocols. The RNA concentration was measured with a NanoDrop ND 1000 spectrometer (NanoDrop Technologies, Wilmington, DE), and the RNA integrity was assessed on an Agilent 2100 Bioanalyzer (Agilent Technologies, Palo Alto, CA). The Low Input Quick Amp Labeling Kit (Agilent Technologies, Santa Clara, CA) was used to amplify and label RNA samples for hybridization according to the manufacturer’s instructions. Two control and two CMS-subjected RASMCs were analyzed with Rat 4 × 44 K Ver.3.0 (Agilent Technologies, Santa Clara, CA). The gene expression analysis method was approved by the committee of Nara Medical University.

### Bioinformatics analysis

The transcriptome data were normalized using Agi4 × 44PreProcess^34^, a package in Bioconductor^35^. Probes that passed four criteria (gIsSaturated, gIsFeatNonUnifOL, gIsPosAndSignif, and gIsWellAboveBG) across the dataset were used for further analysis. A RankProd analysis^36^ was performed to identify differentially expressed genes between RASMCs with and without CMS by calculating the false discovery rate (FDR). DEGs with an FDR < 10% were then converted to human orthologues using the Life Science Knowledge Bank (World Fusion, Tokyo, Japan). The gene symbols of human orthologues were used for further bioinformatics analysis. The identified DEGs are shown in Table S1.

To identify molecules potentially regulating the DEGs and cell processes significantly related to the DEGs, we used Pathway Studio^12^. Pathway Studio uses gene sets derived from natural language processing-based text mining of published literature. The lists of 91 DEGs shown in Table S1 were analyzed with Pathway Studio to predict gene expression regulators and cell processes using subnetwork enrichment analyses of “expression target” and “cell process,” respectively. The predicted expression regulators or cell processes with *p* < 1.00 × 10^−10^ are shown in Table S2 (worksheets 1 and 2).

To identify KEGG pathways related to the DEGs that were found to be potentially regulated by JNK/p38 and related to cell death, we used WebGestalt^13^, a web-based GEne SeT AnaLysis Toolkit, using the subnetwork enrichment analysis for “KEGG.” The predicted KEGG pathways with *p* < 1.00 × 10^−3^ are shown in Table S3.

To identify transcription factors (TFs) for the DEGS, we used iRegulon^14^, which exploits the fact that genes that are co-regulated by the same TFs that commonly share binding sites for the TFs and use gene sets derived from ENCODE ChIP-seq data^37^. We used iRegulon as an application in Cytoscape^37^. The list of the 91 DEGs shown in Table S1 were subjected to iRegulon. The predicted TFs with normalized enrichment scores (NES) > 4 are shown in Table S4.

### Quantitative real-time RT-PCR

Total RNA was extracted using the RNeasy Mini Kit (Qiagen, Valencia, CA) according to the manufacturer’s instructions. For reverse transcription, PrimeScript™ Reverse Transcriptase was used (Takara, Japan) according to the manufacturer’s instructions. The cDNA was resuspended in RNase-free water and used as a template for SYBR Green quantitative real-time PCR using primers designed for selected genes. Real-time PCR was performed using THUNDERBIRD® SYBR qPCR Mix (TOYOBO, Japan). Reactions were performed using the StepOnePlus Real-Time PCR System (Applied Biosystems, Carlsbad, CA). The relative amount of target mRNA normalized to GAPDH was calculated. The quantitative real-time RT-PCR method was approved by the committee of Nara Medical University.

### Immunoblot analysis

Immunoblotting was performed as described previously^10^. Briefly, total protein was separated by sodium dodecyl sulfate polyacrylamide gel electrophoresis (SDS-PAGE) and transferred onto a polyvinylidene difluoride (PVDF) membrane. The membrane was first blocked in Tris-buffered saline with 0.05% Tween 20 (TBS-T) with 5% non-fat skim milk (Nacalai, Japan) and then incubated with the primary antibody. After washing with TBS-T, the membrane was incubated with the secondary antibody in TBS-T with non-fat dry milk. After the wash, the blots were developed with ECL plus Western blotting substrate (Buckinghamshire, UK). Band intensities were quantified by the densitometry of the immunoblots using NIH ImageJ software. The uncropped pictures of immuoblotting are shown in Supplemental Fig. S4. The immunoblot analysis was approved by the committee of Nara Medical University.

### MTT assay

MTT assays were performed as described previously^10^. Briefly, 50 µg of MTT solution (Sigma-Aldrich, St. Louis, MO) was added to the cells, and the cells were then incubated for two hours at 37 °C. Following incubation, the cells were solubilized, and the optical density was measured using a microplate spectrophotometer (Thermo Scientific, Waltham, MA) at 540 nm. The MTT assay method was approved by the committee of Nara Medical University.

### LDH assay

The LDH assay was performed using a Cytotoxicity Detection Kit (Roche Diagnostics, Penzberg, Germany) according to the manufacturer’s instructions. The LDH assay method was approved by the committee of Nara Medical University.

### Abdominal aortic constriction (AAC) protocol

All experimental procedures were approved by the Animal Research Committee of Wakayama Medical University. All animals were housed individually in cages under specific pathogen–free conditions during the experiments. Age- and sex-matched mice were used for the experiments. AAC was performed to induce hypertension. The mice were anesthetized with 1.5% isoflurane, and a longitudinal skin incision was made on the left lateral side of the abdomen. The abdominal aorta was constricted with a 7–0 silk suture tied firmly two times against a 28-gauge blunted needle. After ligation, the needle was quickly removed, the skin was closed, and the mice were allowed to recover. Sham-operated mice underwent a similar surgical procedure without the constriction of the aorta. Six hours after AAC, thoracic aortas were collected from AAC and sham-operated mice and subjected to gene expression and immunohistochemical analyses.

### Immunohistochemical analysis

The aortas were fixed in 4% formaldehyde buffered with PBS (pH 7.2) to prepare paraffin-embedded sections (4 μm thick). The sections were incubated with rabbit anti-CXCL1 polyclonal antibodies (Abcam) and mouse anti-human alpha-SMA monoclonal antibodies (Daco) at 4 °C overnight. Then, the sections were incubated with Cy3-conjugated donkey anti-rabbit IgG polyclonal antibodies and FITC-conjugated donkey anti-mouse IgG polyclonal antibodies. The immunohistochemical analysis method was approved by the committee of Wakayama Medical University.

### Statistical analysis

The Student’s t-test was used to compare differences between two groups, and a one-way ANOVA followed by Tukey’s HSD test was used to compare differences between multiple groups. Differences were considered significant at P < 0.05.

## Electronic supplementary material

Supplementary Dataset 1
