# Supplementary material for: Chemokines protect vascular smooth muscle cells from cell death induced by cyclic mechanical stretch
Source: Sci Rep. 2017 Nov 23;7:16128. doi: 10.1038/s41598-017-15867-8 (PMC5701048; doi:10.1038/s41598-017-15867-8)
Supplement: Supplementary file 1 — Supplementary Dataset 1 [file 41598_2017_15867_MOESM1_ESM.pdf]

Chemokines protect vascular smooth muscle cells from cell death induced by cyclic mechanical stretch

Jing Zhao[1], Yuhei Nishimura[2, 3, 4, 5, 6], Akihiko Kimura[7], Kentaro Ozawa[1, 8], Toshikazu Kondo[7], Toshio Tanaka[3, 4, 5, 6], Masanori Yoshizumi[1]

1. Department of Pharmacology, Nara Medical University School of Medicine, Japan
2. Department of Molecular and Cellular Pharmacology, Pharmacogenomics and Pharmacoinformatics
3. Mie University Medical Zebrafish Research Center
4. Department of Bioinformatics, Mie University Life Science Research Center
5. Department of Omics Medicine, Mie University Industrial Technology Innovation Institute
6. Department of Systems Pharmacology, Mie University Graduate School of Medicine, Tsu, Mie, Japan
7. Department of Forensic Medicine, Wakayama Medical University, Wakayama, Japan
8. Department of Psychiatry, Osaka University Graduate School of Medicine, Osaka, Japan

Corresponding Author: Kentaro Ozawa, M.D., Ph.D.
Department of Pharmacology, Nara Medical University School of Medicine
840 Shijo-cho, Kashihara, Nara, 634–0813, Japan
TEL: +81–744–29–8831
FAX: +81–744–29–0510
E-mail: ko12@naramed-u.ac.jp

Supplemental Informaiton

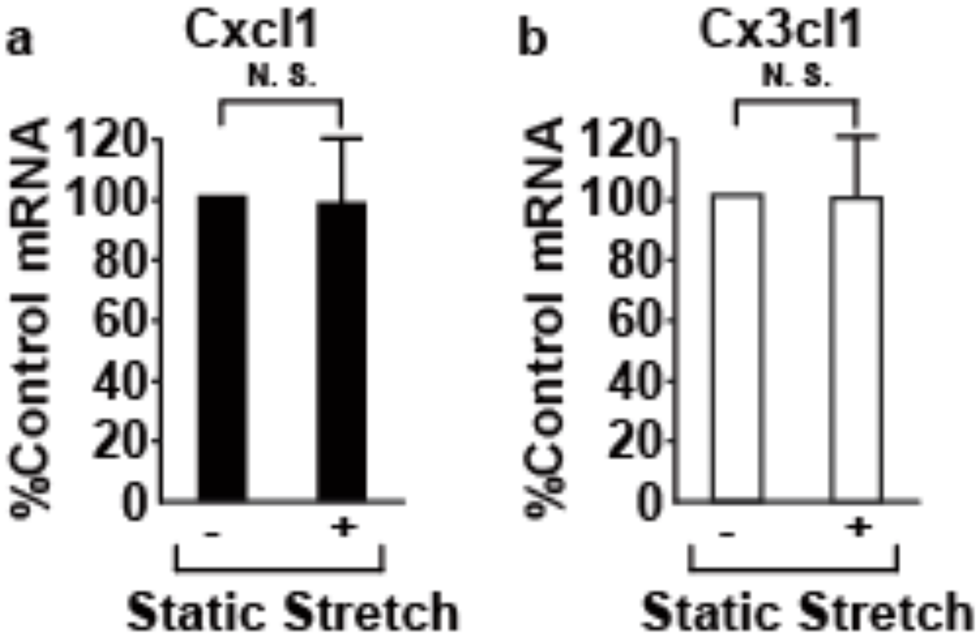

Supplemental Figure S1. Induction of CXCL1 and CX3CL1 in RASMCs subjected to static stretch. RASMCs were subjected to static stretch for four hours. Cells were harvested and analyzed by real-time RT-PCR with specific primers for *Cxcl1* (a) or *Cx3cl1* (b). All values represent means ± SEM (n = 3). N.S. indicates no significant difference.

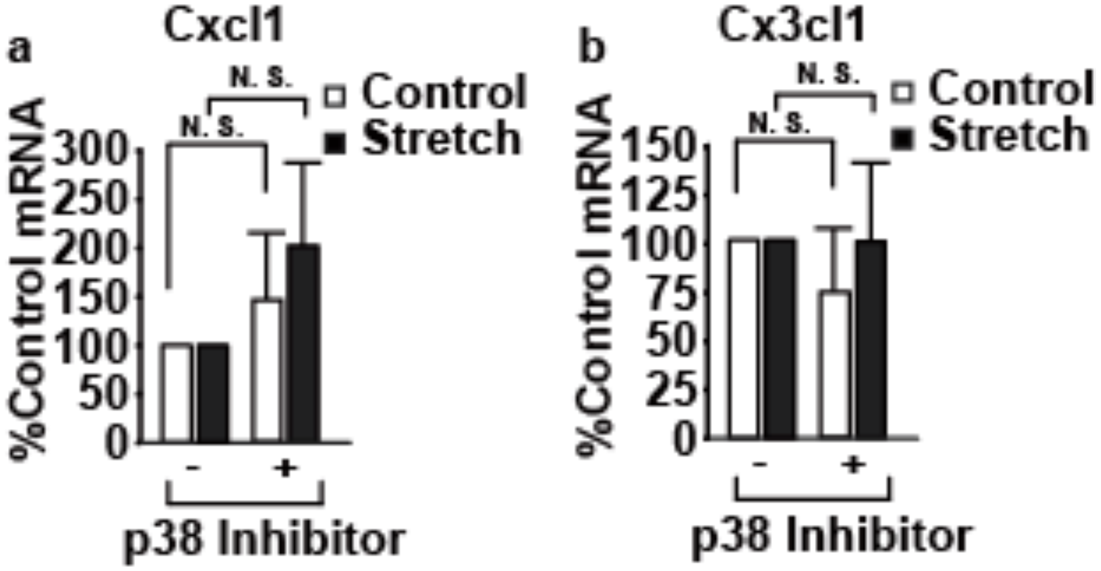

Supplemental Figure S2. Induction of CXCL1 and CX3CL1 in RASMCs subjected to CMS with p38 inhibitor. RASMCs were incubated with SB203580 (20 μM) for 20 min and then subjected to CMS for four hours. Cells were harvested and analyzed by real-time RT-PCR with specific primers for *Cxcl1* (a) or *Cx3cl1* (b). All values represent means ± SEM (n = 3). N.S. indicates no significant difference.

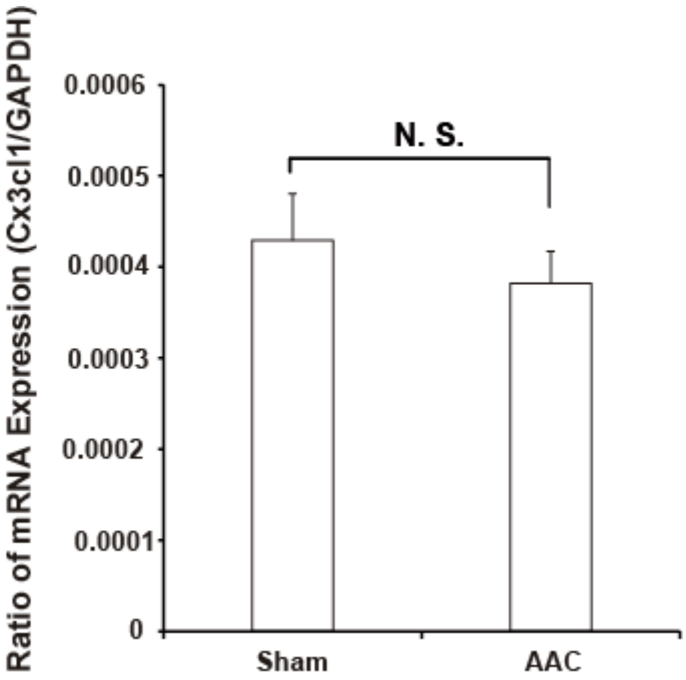

Supplemental Figure S3. AAC-induced CX3CL1 expression in the aorta.

Real-time RT-PCR analysis of *Cx3cl1* gene expression in the aorta of sham and AAC mice 6 h post-operation. All values represent means ± SEM (n = 4–6). N.S. indicates no significant difference.

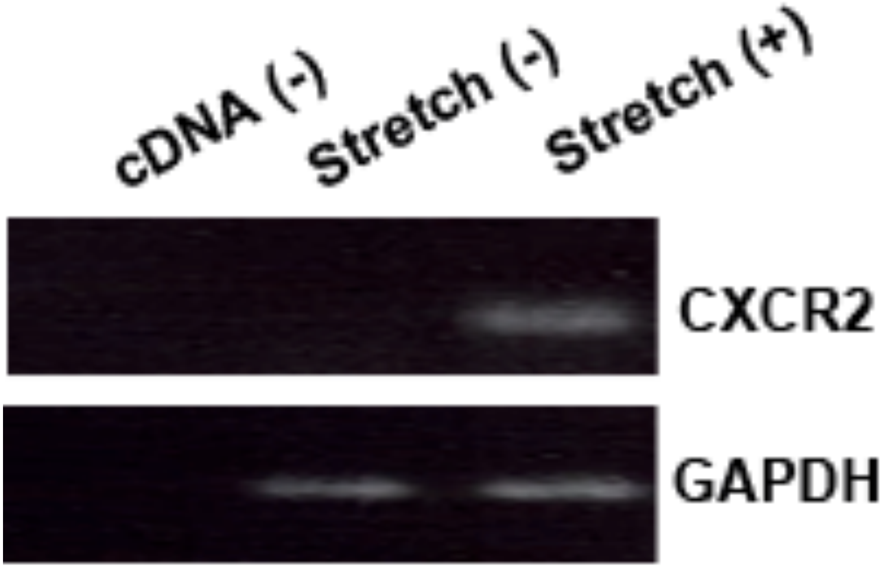

Supplemental Figure S4. Expression of CXCR2 in RASMCs subjected to CMS.

Total RNAs were purified from RASMCs under normal conditions or subjected to CMS for four hours. cDNAs were synthesized from total RNAs, and mock samples (double distilled water) and cDNAs were then amplified using specific primers for CXCR2 (upper panel) and GAPDH (lower panel).

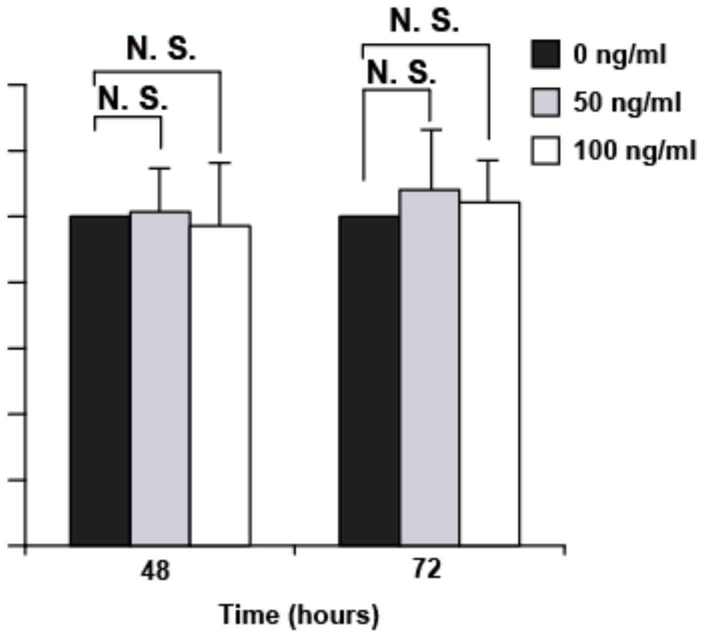

Supplemental Figure S5. Proliferation of RASMCs with recombinant CXCL1.

RASMCs were incubated with recombinant CXCL1 at the indicated concentration and cultured for the indicated times. Cell numbers were evaluated by the MTT assay as described in the Materials and Methods section.

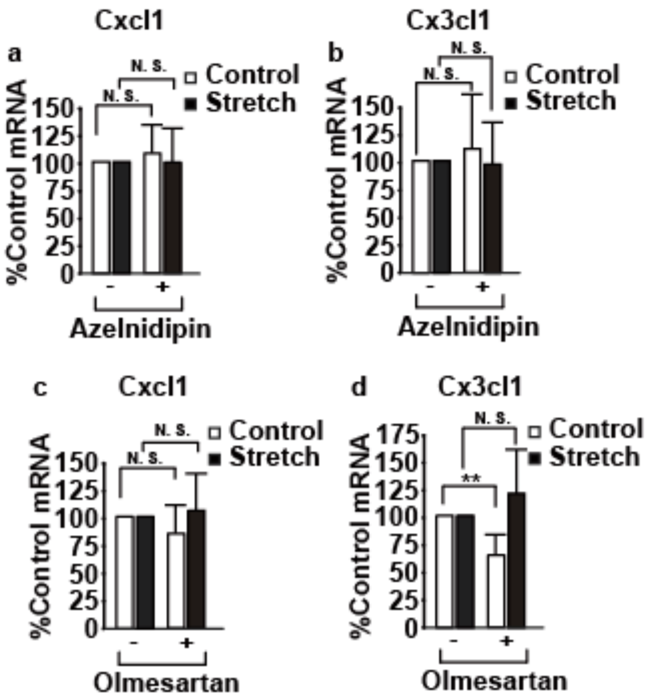

Supplemental Figure S6. Induction of CXCL1 and CX3CL1 in RASMCs subjected to CMS with Azelnidipin or Olmesartan. RASMCs were incubated with Azelnidipin (100 µM) or Olmesartan (100 nM) for 20 min and then subjected to CMS for four hours. Cells were harvested and analyzed by real-time RT-PCR with specific primers for *Cxcl1* (a) or *Cx3cl1* (b). All values represent means ± SEM (n = 3). ** $p < 0.01$ versus control, and N.S. indicates no significant difference.

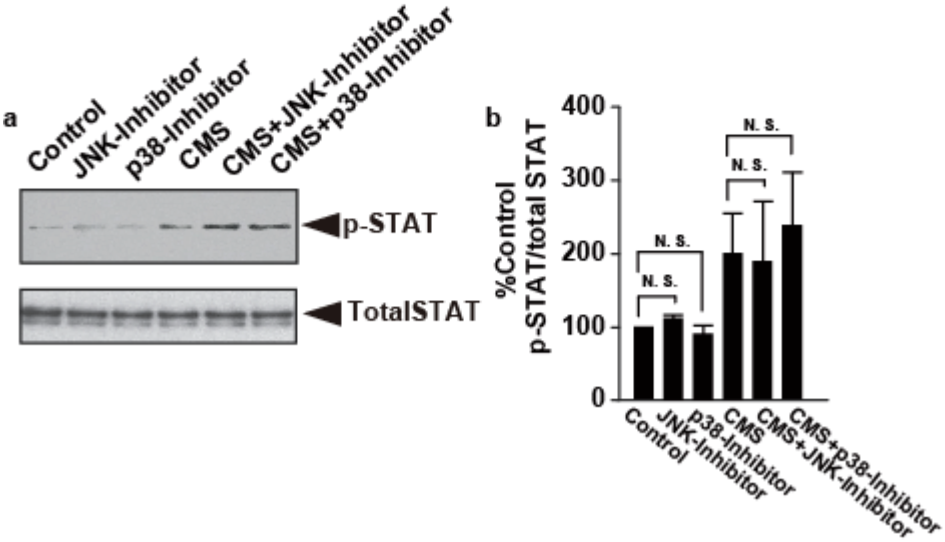

Supplemental Figure S7.

(a) RASMCs were incubated with SP600125 (20 μM) or SB203580 (20 μM) for 20 min and then subjected to CMS for four hours and then harvested. Cell lysates were analyzed by immunoblotting using anti-phosphorylated (upper panel) and total STAT (lower panel) antibodies. (b) The quantity of phosphorylated STAT is expressed as a percentage of the control, normalized with respect to total STAT. Data are means ± SE (n = 3); N.S. indicates no significant difference.

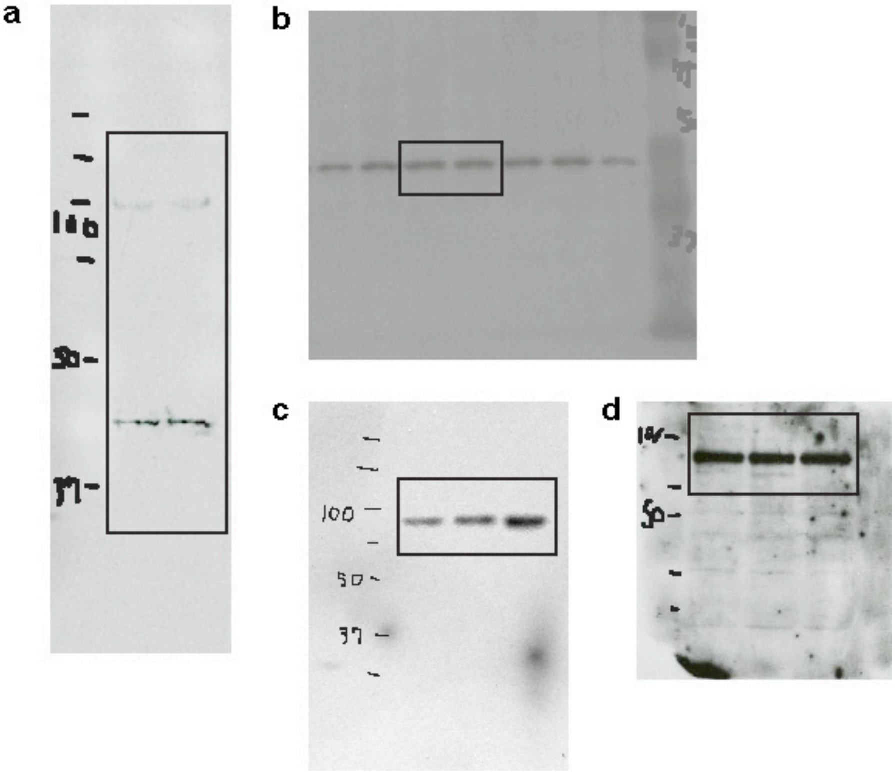

Supplemental Figure S8. Uncropped pictures of immunoblotting.

Uncropped pictures of Fig. 3e (a and b) and Fig. 6b (c and d) were shown.

Table S1 Genes differentially expressed between RASMC with and without cyclic mechanical stretch

Rn symbol	Hs symbol	Gene Name	log (streth/control)	FDR
Abcg1	*ABCG1*	ATP-binding cassette, sub-family G (WHITE), member 1	1.13	0.06
Adh1	*ADH1C*	alcohol dehydrogenase 1C (class I), gamma polypeptide	-1.03	0.09
Adh7	*ADH7*	alcohol dehydrogenase 7 (class IV), mu or sigma polypeptide	-1.09	0.09
Amigo3	*AMIGO3*	adhesion molecule with Ig-like domain 3	1.09	0.06
Apoc2	*APOC2*	apolipoprotein C-II	1.14	0.06
Arl15	*ARL15*	ADP-ribosylation factor-like 15	1.25	0.06
Atf3	*ATF3*	activating transcription factor 3	1.26	0.06
Baalc	*BAALC*	brain and acute leukemia, cytoplasmic	-1.14	0.09
Banp	*BANP*	BTG3 associated nuclear protein	-1.07	0.08
Bdkrb1	*BDKRB1*	bradykinin receptor B1	1.30	0.06
Btg2	*BTG2*	BTG family, member 2	1.10	0.06
C1qtnf3	*C1QTNF3*	C1q and tumor necrosis factor related protein 3	-1.08	0.09
C3	*C3*	complement component 3	1.07	0.09
Ccdc170	*CCDC170*	coiled-coil domain containing 170	1.55	0.06
Ccdc37	*CCDC37*	coiled-coil domain containing 37	-1.11	0.09
Cd180	*CD180*	CD180 molecule	1.14	0.06
Cflar	*CFLAR*	CASP8 and FADD-like apoptosis regulator	1.15	0.06
Cilp	*CILP*	cartilage intermediate layer protein, nucleotide pyrophosphohydrolase	-2.01	0.01
Cx3cl1	*CX3CL1*	chemokine (C-X3-C motif) ligand 1	1.46	0.05
Cxcl1	*CXCL2*	chemokine (C-X-C motif) ligand 2	1.69	0.04
Cxcl2	*CXCL3*	chemokine (C-X-C motif) ligand 3	1.90	0.05
Cyp26b1	*CYP26B1*	cytochrome P450, family 26, subfamily B, polypeptide 1	-1.76	0.08
Cyp7b1	*CYP7B1*	cytochrome P450, family 7, subfamily B, polypeptide 1	1.28	0.06
Ddit3	*DDIT3*	DNA-damage-inducible transcript 3	-1.06	0.08
Dusp5	*DUSP5*	dual specificity phosphatase 5	1.14	0.07
Egln3	*EGLN3*	egl-9 family hypoxia-inducible factor 3	1.49	0.07
Ehbp1	*EHBP1*	EH domain binding protein 1	-1.05	0.09
Eva1a	*EVA1A*	eva-1 homolog A (C. elegans)	1.27	0.05
Fancd2	*FANCD2*	Fanconi anemia, complementation group D2	1.34	0.06
Fcer1g	*FCER1G*	Fc fragment of IgE, high affinity I, receptor for; gamma polypeptide	1.55	0.07
Fgf9	*FGF9*	fibroblast growth factor 9	-1.10	0.09
Galnt14	*GALNT14*	polypeptide N-acetylgalactosaminyltransferase 14	1.08	0.08
Gja5	*GJA5*	gap junction protein, alpha 5, 40kDa	1.19	0.06
H2afy2	*H2AFY2*	H2A histone family, member Y2	-1.48	0.09
Hp	*HP*	haptoglobin	1.19	0.07
Hspa1b	*HSPA1A*	heat shock 70kDa protein 1A	1.79	0.06
Il6	*IL6*	interleukin 6	1.21	0.06
Kcnk7	*KCNK7*	potassium channel, subfamily K, member 7	1.33	0.05
Lamc2	*LAMC2*	laminin, gamma 2	0.99	0.09
Lcn2	*LCN2*	lipocalin 2	1.51	0.06
LOC684871	*C8orf4*	chromosome 8 open reading frame 4	1.17	0.05
LOC691931	*C4orf32*	chromosome 4 open reading frame 32	1.23	0.06
Mapk8ip3	*MAPK8IP3*	mitogen-activated protein kinase 8 interacting protein 3	-1.62	0.09
Mlh3	*MLH3*	mutL homolog 3	-1.14	0.09
Mmp13	*MMP13*	matrix metallopeptidase 13 (collagenase 3)	1.31	0.06
Mmp3	*MMP3*	matrix metallopeptidase 3 (stromelysin 1, progelatinase)	1.82	0.05
Mmp9	*MMP9*	matrix metallopeptidase 9 (gelatinase B, 92kDa gelatinase, 92kDa type IV collagenase)	1.24	0.06
Nfkbiz	*NFKBIZ*	nuclear factor of kappa light polypeptide gene enhancer in B-cells inhibitor, zeta	1.18	0.07
Nos2	*NOS2*	nitric oxide synthase 2, inducible	1.21	0.06
Nr4a1	*NR4A1*	nuclear receptor subfamily 4, group A, member 1	1.11	0.06
Nr4a3	*NR4A3*	nuclear receptor subfamily 4, group A, member 3	1.39	0.05
Nup210	*NUP210*	nucleoporin 210kDa	1.37	0.05
Ogn	*OGN*	osteoglycin	-1.05	0.09
Pcp4	*PCP4*	Purkinje cell protein 4	1.41	0.06
Pde1a	*PDE1A*	phosphodiesterase 1A, calmodulin-dependent	-1.03	0.09
Pde8a	*PDE8A*	phosphodiesterase 8A	1.08	0.06
Pla2g2a	*PLA2G2A*	phospholipase A2, group IIA (platelets, synovial fluid)	1.73	0.05
Plcl1	*PLCL1*	phospholipase C-like 1	-1.45	0.08
Ppm1e	*PPM1E*	protein phosphatase, Mg2+/Mn2+ dependent, 1E	-1.07	0.08
Prg4	*PRG4*	proteoglycan 4	1.16	0.06
Ptges	*PTGES*	prostaglandin E synthase	1.20	0.06
Ptx3	*PTX3*	pentraxin 3, long	1.15	0.07
Rasd1	*RASD1*	RAS, dexamethasone-induced 1	1.16	0.06
Rasl11b	*RASL11B*	RAS-like, family 11, member B	1.25	0.05
Rgs2	*RGS2*	regulator of G-protein signaling 2	1.22	0.06
Rnase10	*RNASE10*	ribonuclease, RNase A family, 10 (non-active)	1.26	0.06
Rnase1l2	*RNASE1*	ribonuclease, RNase A family, 1 (pancreatic)	1.46	0.06
Rnd1	*RND1*	Rho family GTPase 1	1.45	0.06
Sdcbp2	*SDCBP2*	syndecan binding protein (syntenin) 2	1.10	0.06
Selp	*SELP*	selectin P (granule membrane protein 140kDa, antigen CD62)	1.21	0.06
Serpine1	*SERPINE1*	serpin peptidase inhibitor, clade E (nexin, plasminogen activator inhibitor type 1), member 1	1.19	0.06
Sh3bp4	*SH3BP4*	SH3-domain binding protein 4	1.23	0.05
Sh3kbp1	*SH3KBP1*	SH3-domain kinase binding protein 1	1.32	0.09
Slc16a3	*SLC16A3*	solute carrier family 16 (monocarboxylate transporter), member 3	1.38	0.05
Slc45a1	*SLC45A1*	solute carrier family 45, member 1	-1.56	0.09
Slc4a11	*SLC4A11*	solute carrier family 4, sodium borate transporter, member 11	-1.60	0.07
Slc5a3	*SLC5A3*	solute carrier family 5 (sodium/myo-inositol cotransporter), member 3	-1.02	0.09
Spice1	*SPICE1*	spindle and centriole associated protein 1	-1.02	0.09
Star	*STAR*	steroidogenic acute regulatory protein	1.13	0.06
Steap4	*STEAP4*	STEAP family member 4	1.19	0.06
Tbc1d9	*TBC1D9*	TBC1 domain family, member 9 (with GRAM domain)	1.13	0.06
Tfpi2	*TFPI2*	tissue factor pathway inhibitor 2	1.50	0.07
Tfrc	*TFRC*	transferrin receptor	1.21	0.06
Tgm1	*TGM1*	transglutaminase 1	1.30	0.05
Tlr2	*TLR2*	toll-like receptor 2	1.26	0.06
Tnfrsf12a	*TNFRSF12A*	tumor necrosis factor receptor superfamily, member 12A	1.06	0.07
Tnfrsf9	*TNFRSF9*	tumor necrosis factor receptor superfamily, member 9	1.21	0.06
Tnip3	*TNIP3*	TNFAIP3 interacting protein 3	1.28	0.05
Uap1	*UAP1*	UDP-N-acetylglucosamine pyrophosphorylase 1	1.15	0.07
Xkr6	*XKR6*	XK, Kell blood group complex subunit-related family, member 6	1.65	0.06
Zc3h12a	*ZC3H12A*	zinc finger CCCH-type containing 12A	1.36	0.06

Table S2-1 Gene expression regulators for the 91 DEG in RASMC treated with cyclic mechanical stretch

Gene expression regulator	p value	Overlapping Entities
TNF	4.88E-21	GJA5;MMP13;CYP7B1;PTGES;CXCL2;TNFRSF9;TNFRSF12A;NFKBIZ;PTX3;NR4A3;ATF3;EGLN3;HP;NUP210;RGS2;PLA2G2A;STAR;BTG2;STEAP4;MMP9;ZC3H12A;MMP3;TNIP3;ABCG1;TFRC;SLC5A3;TGM1;LCN2;NR4A1;PRG4;SERPINE1;CFLAR;SELP;FGF9;CX3CL1;DDIT3;BDKRB1;TFPI2;RNASE1;FCER1G;NOS2;APOC2;TLR2;C3;CXCL3;HSPA1A;IL6
NFKBI	1.19E-19	MMP3;MMP13;CXCL2;PTGES;LCN2;ATF3;EGLN3;NR4A1;SERPINE1;CFLAR;CX3CL1;DDIT3;SELP;TFPI2;BDKRB1;NOS2;TLR2;PLA2G2A;STEAP4;MMP9;CXCL3;IL6
IL1 family	1.63E-19	MMP13;CYP7B1;CXCL2;PTGES;NFKBIZ;PTX3;ATF3;HP;PLA2G2A;STAR;MMP9;ZC3H12A;MMP3;TFRC;LCN2;NR4A1;SERPINE1;PRG4;DDIT3;SELP;FGF9;CX3CL1;BDKRB1;NOS2;APOC2;TLR2;C3;HSPA1A;IL6
IL1B	1.03E-18	LAMC2;MMP13;PTGES;CXCL2;TNFRSF9;TNFRSF12A;NFKBIZ;PTX3;NR4A3;HP;EGLN3;RGS2;PLA2G2A;STAR;STEAP4;MMP9;ZC3H12A;MMP3;ABCG1;CYP26B1;TFRC;LCN2;NR4A1;PRG4;SERPINE1;SELP;DDIT3;CX3CL1;TFPI2;BDKRB1;RNASE1;NOS2;TLR2;C3;HSPA1A;CXCL3;IL6
**MAPK14**	**1.04E-17**	**MMP13;CXCL2;PTGES;NFKBIZ;PTX3;NR4A3;ATF3;EGLN3;RGS2;PLA2G2A;STAR;MMP9;ZC3H12A;MMP3;TFRC;SLC5A3;RND1;LCN2;NR4A1;SERPINE1;DDIT3;SELP;CX3CL1;BDKRB1;NOS2;TLR2;CXCL3;HSPA1A;IL6**
cytokine	3.53E-17	LAMC2;MMP13;PTGES;CXCL2;TNFRSF12A;PTX3;NR4A3;HP;EGLN3;RGS2;PLA2G2A;STAR;STEAP4;MMP9;BAALC;ZC3H12A;MMP3;ABCG1;CYP26B1;TFRC;TGM1;LCN2;NR4A1;SERPINE1;PRG4;PDE1A;CFLAR;CX3CL1;SELP;DDIT3;BDKRB1;RNASE1;NOS2;C3;OGN;IL6
NF-kB	6.93E-17	MMP13;CXCL2;PTGES;TNFRSF9;NFKBIZ;PTX3;NR4A3;ATF3;FANCD2;PLA2G2A;BTG2;STEAP4;MMP9;BAALC;ZC3H12A;TNIP3;MMP3;ABCG1;TFRC;SLC5A3;TGM1;LCN2;NR4A1;SERPINE1;PDE1A;CFLAR;DDIT3;CX3CL1;SELP;TFPI2;BDKRB1;NOS2;TLR2;C3;HSPA1A;CXCL3;IL6
oxidized LDL	3.85E-16	MMP3;ABCG1;GJA5;MMP13;CXCL2;PTGES;TNFRSF9;PTX3;NR4A3;NR4A1;SERPINE1;CFLAR;CX3CL1;SELP;DDIT3;BDKRB1;NOS2;PLA2G2A;TLR2;STAR;MMP9;HSPA1A;IL6
TLR	4.59E-16	TNIP3;MMP3;PTGES;CXCL2;NFKBIZ;LCN2;PTX3;ATF3;HP;SERPINE1;CFLAR;SELP;CX3CL1;DDIT3;NOS2;TLR2;C3;STEAP4;MMP9;HSPA1A;ZC3H12A;IL6
PI3K	4.75E-16	GJA5;LAMC2;MMP13;PTGES;CXCL2;CYP7B1;TNFRSF9;PTX3;ATF3;EGLN3;SLC16A3;RGS2;PLA2G2A;STAR;BTG2;MMP9;ZC3H12A;MMP3;ABCG1;TFRC;LCN2;NR4A1;SERPINE1;CFLAR;SELP;DDIT3;BDKRB1;NOS2;C3;HSPA1A;IL6
IL1A	8.66E-16	MMP3;LAMC2;MMP13;CXCL2;PTGES;TGM1;LCN2;NFKBIZ;PRG4;SERPINE1;CX3CL1;DDIT3;SELP;NOS2;TLR2;C3;STAR;MMP9;CXCL3;HSPA1A;IL6
Inflammatory Cytokines	1.32E-15	MMP13;PTGES;CXCL2;TNFRSF9;TNFRSF12A;PTX3;EGLN3;HP;PLA2G2A;STAR;STEAP4;MMP9;MMP3;ABCG1;TFRC;LCN2;SERPINE1;PRG4;CFLAR;CX3CL1;SELP;DDIT3;BDKRB1;NOS2;C3;CXCL3;IL6
TLR2	1.51E-15	MMP3;ABCG1;MMP13;PTGES;CXCL2;NFKBIZ;LCN2;PTX3;ATF3;NR4A1;RGS2;SELP;DDIT3;NOS2;TLR2;STAR;MMP9;HSPA1A;IL6;ZC3H12A
mitogen-activated protein kinase	2.26E-15	LAMC2;MMP13;CXCL2;CYP7B1;PTGES;TNFRSF9;TNFRSF12A;NR4A3;ATF3;HP;PLA2G2A;STAR;BTG2;STEAP4;MMP9;ZC3H12A;MMP3;ABCG1;TGM1;DUSP5;LCN2;NR4A1;SERPINE1;CFLAR;CX3CL1;SELP;DDIT3;TFPI2;NOS2;HSPA1A;IL6
**MAPK8**	**3.24E-15**	**LAMC2;MMP13;CXCL2;PTGES;TNFRSF9;TNFRSF12A;PTX3;ATF3;STAR;MMP9;MMP3;ABCG1;TFRC;TGM1;LCN2;NR4A1;SERPINE1;CFLAR;SELP;DDIT3;BDKRB1;NOS2;TLR2;CXCL3;HSPA1A;IL6**
PKC	4.13E-15	LAMC2;MMP13;PTGES;CXCL2;ATF3;NR4A3;RGS2;PLA2G2A;BTG2;STAR;MMP9;MMP3;ABCG1;TFRC;TGM1;NR4A1;SERPINE1;CFLAR;FGF9;DDIT3;CX3CL1;SELP;BDKRB1;NOS2;TLR2;C3;HSPA1A;IL6
CEBPB	5.24E-15	MMP3;ABCG1;MMP13;PTGES;CXCL2;LCN2;PTX3;ATF3;BANP;HP;NR4A1;FGF9;DDIT3;BDKRB1;NOS2;PLA2G2A;STAR;C3;BTG2;STEAP4;MMP9;HSPA1A;IL6
TLR4	6.65E-15	MMP3;LAMC2;ABCG1;MMP13;PTGES;CXCL2;LCN2;PTX3;ATF3;NR4A1;RGS2;SERPINE1;CX3CL1;DDIT3;SELP;NOS2;TLR2;C3;MMP9;HSPA1A;IL6;ZC3H12A
HDL	8.84E-15	MMP3;ABCG1;PTX3;ATF3;SERPINE1;CFLAR;DDIT3;SELP;CX3CL1;NOS2;TLR2;C3;STAR;MMP9;IL6
MAPK3	1.10E-14	MMP13;PTGES;CXCL2;PTX3;NR4A3;ATF3;RGS2;PLA2G2A;STAR;MMP9;MMP3;SH3KBP1;LCN2;DUSP5;NR4A1;SERPINE1;DDIT3;TFPI2;GALNT14;NOS2;C3;HSPA1A;IL6;OGN
F2	1.31E-14	MMP3;TFRC;MMP13;CXCL2;PTGES;LCN2;NR4A1;RGS2;SERPINE1;DDIT3;SELP;CX3CL1;TFPI2;NOS2;PLA2G2A;STAR;MMP9;CXCL3;HSPA1A;IL6
IL10	1.84E-14	TNIP3;MMP3;ABCG1;MMP13;TFRC;PTGES;CXCL2;TNFRSF9;NFKBIZ;LCN2;PTX3;SERPINE1;CFLAR;SELP;NOS2;PLA2G2A;STAR;C3;MMP9;CXCL3;IL6
PDGF	2.83E-14	MMP3;GJA5;TFRC;MMP13;CXCL2;PTGES;LCN2;TNFRSF12A;NR4A3;NR4A1;RGS2;PRG4;SERPINE1;SELP;FGF9;NOS2;PLA2G2A;STAR;BTG2;MMP9;HSPA1A;ZC3H12A;IL6
TLR3	3.20E-14	MMP3;ABCG1;MMP13;PTGES;TGM1;PTX3;ATF3;NR4A1;RGS2;CX3CL1;DDIT3;BDKRB1;NOS2;TLR2;MMP9;HSPA1A;IL6
EDN1	3.21E-14	MMP3;GJA5;MMP13;PTGES;TNFRSF12A;ATF3;EGLN3;RGS2;SERPINE1;SELP;CX3CL1;BDKRB1;NOS2;PLA2G2A;TLR2;STAR;MMP9;HSPA1A;IL6
SP1	3.34E-14	LAMC2;GJA5;MMP13;PTGES;CXCL2;CYP7B1;TNFRSF12A;PTX3;RGS2;PLA2G2A;BTG2;STAR;MMP9;BAALC;MMP3;ABCG1;TFRC;TGM1;LCN2;BANP;NR4A1;ADH1C;SERPINE1;CFLAR;C1QTNF3;DDIT3;CX3CL1;SELP;TFPI2;NOS2;FCER1G;TLR2;HSPA1A;CXCL3;IL6
**MAPK9**	**3.86E-14**	**MMP3;MMP13;CXCL2;PTGES;TNFRSF12A;PTX3;ATF3;NR4A1;SERPINE1;CFLAR;NOS2;TLR2;MMP9;IL6**
IL17A	6.81E-14	MMP3;MMP13;CXCL2;PTGES;NFKBIZ;LCN2;PTX3;CX3CL1;DDIT3;SELP;NOS2;PLA2G2A;C3;STEAP4;MMP9;CXCL3;ZC3H12A;IL6
ADIPOQ	7.54E-14	MMP3;TNIP3;ABCG1;MMP13;CXCL2;PTGES;LCN2;PTX3;SERPINE1;SELP;NOS2;TLR2;C3;STAR;STEAP4;MMP9;CXCL3;IL6
MAPK1	8.13E-14	LAMC2;MMP13;PTGES;CXCL2;PTX3;ATF3;NR4A3;PLA2G2A;STAR;MMP9;MMP3;ABCG1;LCN2;DUSP5;NR4A1;SERPINE1;CFLAR;DDIT3;FGF9;CX3CL1;SELP;TFPI2;GALNT14;NOS2;TLR2;C3;HSPA1A;OGN;IL6
growth factor	1.93E-13	GJA5;MMP13;PTGES;TNFRSF12A;PTX3;ATF3;NR4A3;HP;PLA2G2A;BTG2;STAR;MMP9;MMP3;TFRC;LCN2;NR4A1;PRG4;SERPINE1;CFLAR;CX3CL1;DDIT3;BDKRB1;NOS2;HSPA1A;OGN;IL6
HIF1A	1.99E-13	MMP13;PTGES;TNFRSF12A;PTX3;NR4A3;EGLN3;HP;SLC16A3;NUP210;BTG2;STAR;MMP9;MMP3;TFRC;LCN2;NR4A1;SERPINE1;PRG4;DDIT3;NOS2;TLR2;HSPA1A;CXCL3;IL6
EGF	3.12E-13	GJA5;LAMC2;MMP13;PTGES;CXCL2;TNFRSF12A;PTX3;NR4A3;ATF3;EGLN3;RGS2;STAR;MMP9;MMP3;TFRC;TGM1;LCN2;DUSP5;NR4A1;SERPINE1;DDIT3;BDKRB1;TFPI2;NOS2;HSPA1A;IL6
JUN	3.57E-13	MMP3;ABCG1;MMP13;CXCL2;TNFRSF9;TNFRSF12A;DUSP5;LCN2;PTX3;ATF3;NR4A1;SERPINE1;CFLAR;DDIT3;CX3CL1;C1QTNF3;BDKRB1;NOS2;STAR;MMP9;CXCL3;HSPA1A;IL6
AGTR1	3.84E-13	MMP3;GJA5;MMP13;PTGES;ATF3;NR4A1;RGS2;SERPINE1;SELP;DDIT3;NOS2;PLA2G2A;TLR2;STAR;MMP9;HSPA1A;IL6
SCARB1	4.68E-13	MMP3;CX3CL1;NOS2;PTX3;TLR2;STAR;MMP9;SERPINE1;IL6
Jun/Fos	5.09E-13	GJA5;LAMC2;MMP13;PTGES;CXCL2;TNFRSF9;TNFRSF12A;PTX3;ATF3;PLA2G2A;STAR;MMP9;MMP3;TFRC;TGM1;LCN2;NR4A1;SERPINE1;CFLAR;C1QTNF3;SELP;CX3CL1;DDIT3;TFPI2;NOS2;HSPA1A;IL6
IL22	5.32E-13	MMP3;ABCG1;MMP13;CXCL2;LCN2;PTX3;HP;DDIT3;NOS2;PLA2G2A;C3;MMP9;CXCL3;IL6
IFNG	6.96E-13	MMP13;PTGES;CXCL2;TNFRSF12A;NFKBIZ;PTX3;ATF3;EGLN3;HP;PLA2G2A;STAR;MMP9;MMP3;ABCG1;TFRC;TGM1;LCN2;SERPINE1;CFLAR;CX3CL1;SELP;DDIT3;BDKRB1;NOS2;FCER1G;TLR2;C3;CXCL3;HSPA1A;OGN;IL6
NAD(P)H oxidase	8.24E-13	MMP13;PTGES;CXCL2;SLC5A3;LCN2;ATF3;SERPINE1;CX3CL1;SELP;NOS2;TLR2;PLA2G2A;MMP9;IL6
histone deacetylase	9.85E-13	MMP13;CYP7B1;PTGES;CXCL2;ATF3;NR4A3;EGLN3;STAR;STEAP4;MMP9;ZC3H12A;MMP3;ABCG1;LCN2;DUSP5;NR4A1;SERPINE1;CFLAR;DDIT3;CX3CL1;TFPI2;NOS2;TLR2;HSPA1A;IL6
IgG	1.02E-12	MMP3;MMP13;TFRC;CXCL2;NFKBIZ;ATF3;NR4A3;SERPINE1;CX3CL1;SELP;NOS2;STAR;MMP9;IL6
IL1RN	1.05E-12	MMP3;LAMC2;MMP13;CXCL2;NFKBIZ;HP;PRG4;BDKRB1;NOS2;TLR2;PLA2G2A;MMP9;IL6
prostaglandin-endoperoxide synthas	2.24E-12	MMP3;CXCL2;LCN2;ATF3;RGS2;SERPINE1;SELP;NOS2;STAR;MMP9;HSPA1A;IL6
SPHK1	2.74E-12	DDIT3;SELP;MMP13;TFRC;PTGES;CXCL2;NOS2;PTX3;MMP9;IL6;SERPINE1
CD36	3.33E-12	SELP;DDIT3;ABCG1;CXCL2;NOS2;ATF3;MMP9;HSPA1A;SERPINE1;IL6
PTGS2	3.70E-12	MMP3;MMP13;CXCL2;PTGES;LCN2;RGS2;PRG4;SERPINE1;CFLAR;DDIT3;TFPI2;NOS2;STAR;MMP9;HSPA1A;IL6
XDH	3.83E-12	SELP;DDIT3;ABCG1;MMP13;NOS2;STAR;MMP9;HSPA1A;SERPINE1;IL6
IL17RA	6.74E-12	MMP3;CX3CL1;MMP13;CXCL2;NOS2;NFKBIZ;LCN2;MMP9;IL6
interleukin	7.03E-12	MMP3;MMP13;TFRC;CXCL2;PTX3;SERPINE1;CX3CL1;SELP;BDKRB1;NOS2;MMP9;CXCL3;IL6
TLR9	7.15E-12	MMP13;PTGES;CXCL2;NFKBIZ;ATF3;SERPINE1;CFLAR;NOS2;TLR2;CD180;MMP9;HSPA1A;IL6;ZC3H12A
LDL	7.18E-12	MMP3;ABCG1;GJA5;CXCL2;PTX3;NR4A3;NR4A1;SERPINE1;SELP;NOS2;C3;STAR;MMP9;HSPA1A;CXCL3;IL6
VEGFA	9.21E-12	MMP3;GJA5;MMP13;PTGES;TNFRSF9;DUSP5;TNFRSF12A;NR4A3;ATF3;NR4A1;SERPINE1;CFLAR;DDIT3;CX3CL1;TFPI2;NOS2;STAR;MMP9;OGN
beta adrenoceptor	1.81E-11	MMP3;TFRC;MMP13;NR4A3;NR4A1;RGS2;CX3CL1;DDIT3;NOS2;MMP9;CXCL3;HSPA1A;IL6
NFKBIA	2.11E-11	MMP3;CFLAR;CX3CL1;MMP13;PTGES;NOS2;LCN2;ATF3;NR4A3;MMP9;IL6
MAP2K3	2.49E-11	MMP3;DDIT3;MMP13;NOS2;TLR2;ATF3;MMP9;SERPINE1;IL6
AGER	2.50E-11	MMP3;LAMC2;ABCG1;MMP13;CXCL2;LCN2;SERPINE1;SELP;NOS2;TLR2;MMP9;IL6
PTGER4	2.50E-11	MMP3;MMP13;PTGES;CXCL2;NR4A1;SERPINE1;CX3CL1;SELP;NOS2;STAR;MMP9;IL6
ALB	2.51E-11	ABCG1;TFRC;PTGES;CXCL2;LCN2;RGS2;SERPINE1;CFLAR;CX3CL1;NOS2;TLR2;APOC2;MMP9;IL6
HGF	2.63E-11	MMP3;LAMC2;MMP13;CXCL2;PTGES;LCN2;TNFRSF12A;ATF3;HP;NR4A1;SERPINE1;CFLAR;NOS2;TLR2;STAR;C3;MMP9;IL6
CEBPA	2.69E-11	MMP13;CXCL2;PTGES;TNFRSF12A;LCN2;PTX3;ATF3;HP;NR4A1;ADH1C;SERPINE1;CFLAR;DDIT3;FCER1G;NOS2;PLA2G2A;STAR;C3;STEAP4;HSPA1A;IL6
LXR	4.25E-11	MMP3;ABCG1;PTGES;CYP7B1;CXCL2;TGM1;ATF3;HP;SELP;NOS2;PLA2G2A;APOC2;C3;STAR;MMP9;IL6
HMOX1	4.27E-11	MMP3;ABCG1;MMP13;TFRC;CXCL2;PTGES;SERPINE1;SELP;DDIT3;NOS2;MMP9;HSPA1A;CXCL3;IL6
JAK	4.30E-11	MMP3;MMP13;LCN2;PTX3;ATF3;SERPINE1;CX3CL1;SELP;NOS2;PLA2G2A;TLR2;STAR;MMP9;HSPA1A;IL6
AMPK	6.33E-11	MMP3;ABCG1;MMP13;CXCL2;NR4A3;SLC16A3;NR4A1;SERPINE1;CFLAR;DDIT3;NOS2;PLA2G2A;STAR;MMP9;HSPA1A;IL6
PLCG1	7.72E-11	MMP3;DDIT3;MMP13;NOS2;MMP9;NR4A1;HSPA1A;SERPINE1;IL6
TNFRSF1A	7.74E-11	CFLAR;MMP3;CX3CL1;CXCL2;NOS2;LCN2;MMP9;NR4A1;HSPA1A;SERPINE1;IL6
WNT5A	7.77E-11	MMP3;LAMC2;ABCG1;MMP13;CXCL2;LCN2;CX3CL1;FGF9;NOS2;STAR;MMP9;IL6
PPARG	8.70E-11	MMP3;ABCG1;CYP26B1;MMP13;PTGES;TGM1;LCN2;HP;EGLN3;SERPINE1;CFLAR;CX3CL1;SELP;DDIT3;NOS2;PLA2G2A;TLR2;STAR;STEAP4;MMP9;HSPA1A;IL6
CRP	9.49E-11	MMP3;ABCG1;TFRC;CXCL2;SERPINE1;SELP;DDIT3;NOS2;TLR2;BTG2;C3;MMP9
HMGB1	9.74E-11	MMP3;MMP13;CXCL2;PTGES;PTX3;SERPINE1;SELP;NOS2;PLA2G2A;TLR2;MMP9;CXCL3;IL6

Table S2-2 Cell processes significantly related to the 91 DEG in RASMC treated with cyclic mechanical stretch

Cell process	p value	Overlapping Entities
inflammatory response	1.58E-19	MMP13;CXCL2;PTGES;PCP4;TNFRSF9;TNFRSF12A;NFKBIZ;PTX3;ATF3;NR4A3;HP;SLC16A3;PLA2G2A;BTG2;STAR;CD180;STEAP4;MMP9;ZC3H12A;TNIP3;MMP3;ABCG1;TFRC;LCN2;BANP;NR4A1;SERPINE1;CFLAR;CX3CL1;C1QTNF3;FGF9;SELP;DDIT3;TFPI2;BDKRB1;RNASE1;FCER1G;NOS2;TLR2;APOC2;C3;CXCL3;HSPA1A;IL6
SMC proliferation	6.51E-17	MMP13;PTGES;TNFRSF9;TNFRSF12A;PTX3;NR4A3;SLC16A3;CD180;MMP9;ZC3H12A;MMP3;CYP26B1;LCN2;NR4A1;PDE1A;SERPINE1;DDIT3;FGF9;CX3CL1;C1QTNF3;SELP;TFPI2;BDKRB1;NOS2;TLR2;HSPA1A;IL6
immune response	1.23E-14	LAMC2;MMP13;CXCL2;PTGES;TNFRSF9;TNFRSF12A;PTX3;ATF3;HP;EGLN3;NUP210;FANCD2;SH3BP4;PLA2G2A;CD180;STEAP4;MMP9;ZC3H12A;MMP3;UAP1;TFRC;SH3KBP1;RASL11B;LCN2;BANP;NR4A1;PRG4;SERPINE1;CFLAR;DDIT3;CX3CL1;SELP;BDKRB1;NOS2;FCER1G;TLR2;C3;HSPA1A;CXCL3;IL6
cell infiltration	1.58E-14	MMP3;GJA5;MMP13;TFRC;CXCL2;TNFRSF9;TNFRSF12A;NFKBIZ;LCN2;PTX3;ATF3;SERPINE1;DDIT3;SELP;CX3CL1;BDKRB1;NOS2;TLR2;BTG2;MMP9;IL6
neuron apoptosis	2.46E-14	MMP3;TFRC;SH3KBP1;ATF3;EGLN3;SERPINE1;FGF9;DDIT3;CX3CL1;BDKRB1;NOS2;MAPK8IP3;TLR2;PLA2G2A;MMP9;HSPA1A;ZC3H12A;IL6
Angiogenesis	4.32E-14	GJA5;LAMC2;MMP13;CXCL2;PTGES;TNFRSF9;TNFRSF12A;PTX3;ATF3;NR4A3;EGLN3;HP;RGS2;PLA2G2A;CD180;MMP9;ZC3H12A;MMP3;ABCG1;CYP26B1;TFRC;LCN2;MLH3;NR4A1;SERPINE1;CFLAR;CX3CL1;SELP;C1QTNF3;DDIT3;FGF9;TFPI2;BDKRB1;RNASE1;NOS2;TLR2;APOC2;C3;CXCL3;HSPA1A;OGN;IL6
macrophage apoptosis	4.78E-14	ABCG1;PTX3;ATF3;EGLN3;NR4A1;CFLAR;CX3CL1;DDIT3;TFPI2;NOS2;TLR2;STEAP4;MMP9;HSPA1A;ZC3H12A;IL6
ROS generation	7.88E-14	GJA5;CXCL2;PTGES;TNFRSF9;TNFRSF12A;ATF3;HP;PLA2G2A;BTG2;STEAP4;MMP9;ZC3H12A;MMP3;ABCG1;TFRC;LCN2;SERPINE1;CFLAR;SELP;C1QTNF3;DDIT3;CX3CL1;BDKRB1;RNASE1;FCER1G;NOS2;TLR2;APOC2;C3;HSPA1A;OGN;IL6
neuronal death	2.88E-13	PTGES;CXCL2;PCP4;TNFRSF12A;PTX3;ATF3;NR4A3;EGLN3;MAPK8IP3;PLA2G2A;MMP9;MMP3;SH3KBP1;LCN2;NR4A1;SERPINE1;FGF9;DDIT3;SELP;CX3CL1;BDKRB1;FCER1G;NOS2;TLR2;C3;HSPA1A;IL6;RASD1
lipid storage	3.87E-13	MMP13;TNFRSF12A;PTX3;NR4A3;EGLN3;RGS2;FANCD2;PLA2G2A;STAR;BTG2;STEAP4;MMP9;MMP3;ABCG1;LCN2;NR4A1;SERPINE1;CFLAR;DDIT3;C1QTNF3;BDKRB1;NOS2;TLR2;C3;HSPA1A;IL6;RASD1
cell damage	4.86E-13	SLC45A1;PTGES;CXCL2;SLC4A11;TNFRSF12A;PTX3;ATF3;NR4A3;HP;PLA2G2A;STAR;MMP9;TFRC;TGM1;LCN2;SERPINE1;CFLAR;DDIT3;CX3CL1;SELP;NOS2;TLR2;HSPA1A;IL6;RASD1
cell differentiation	5.06E-13	GJA5;LAMC2;CXCL2;TNFRSF9;TNFRSF12A;PTX3;HP;EGLN3;RGS2;SDCBP2;MAPK8IP3;PLA2G2A;STAR;STEAP4;H2AFY2;TNIP3;MMP3;CYP26B1;SH3KBP1;SLC5A3;TGM1;DUSP5;LCN2;PDE8A;CFLAR;CX3CL1;NOS2;TLR2;HSPA1A;OGN;MMP13;PTGES;NFKBIZ;ATF3;NR4A3;NUP210;FANCD2;BTG2;CD180;MMP9;BAALC;ZC3H12A;UAP1;ABCG1;TFRC;EVA1A;BANP;NR4A1;PRG4;SERPINE1;C1QTNF3;FGF9;DDIT3;SELP;BDKRB1;TFPI2;FCER1G;APOC2;C3;IL6;RASD1
kidney function	5.66E-13	SH3KBP1;CYP7B1;PTGES;CXCL2;LCN2;TNFRSF12A;HP;NR4A1;RGS2;SERPINE1;SELP;DDIT3;BDKRB1;NOS2;TLR2;PLA2G2A;C3;CD180;MMP9;HSPA1A;IL6
blood clotting	7.34E-13	MMP3;PTGES;LCN2;PTX3;NR4A3;HP;NR4A1;SERPINE1;CX3CL1;SELP;TFPI2;RNASE1;NOS2;FCER1G;PLA2G2A;C3;MMP9;HSPA1A;IL6
leukocyte migration	1.78E-12	MMP3;ABCG1;MMP13;CXCL2;TNFRSF9;LCN2;PTX3;ATF3;SERPINE1;CX3CL1;SELP;BDKRB1;NOS2;TLR2;MMP9;CXCL3;IL6
cell growth	2.06E-12	LAMC2;GJA5;CXCL2;CYP7B1;TNFRSF9;SLC4A11;TNFRSF12A;EGLN3;HP;RGS2;ADH7;SH3BP4;PLA2G2A;STEAP4;MMP3;CYP26B1;RND1;DUSP5;LCN2;PDE1A;CFLAR;CX3CL1;NOS2;EHBP1;TLR2;HSPA1A;OGN;MMP13;PTGES;NFKBIZ;ATF3;NR4A3;FANCD2;BTG2;CD180;MMP9;ABCG1;TFRC;BANP;NR4A1;SERPINE1;PRG4;C1QTNF3;SELP;DDIT3;FGF9;TFPI2;BDKRB1;RNASE1;APOC2;IL6;RASD1
cell proliferation	2.16E-12	LAMC2;GJA5;CXCL2;CYP7B1;SLC4A11;TNFRSF9;TNFRSF12A;PTX3;HP;EGLN3;RGS2;SDCBP2;SH3BP4;MAPK8IP3;PLA2G2A;STAR;STEAP4;H2AFY2;MMP3;CYP26B1;SH3KBP1;TGM1;CILP;DUSP5;LCN2;PDE1A;CFLAR;CX3CL1;C8orf4;NOS2;TLR2;CXCL3;HSPA1A;OGN;MMP13;PTGES;PCP4;NFKBIZ;NR4A3;ATF3;SLC16A3;NUP210;FANCD2;BTG2;CD180;MMP9;BAALC;ZC3H12A;UAP1;ABCG1;TFRC;EVA1A;NR4A1;PRG4;SERPINE1;DDIT3;SELP;C1QTNF3;FGF9;TFPI2;BDKRB1;GALNT14;RNASE1;FCER1G;C3;IL6;RASD1
smooth muscle cell migration	4.24E-12	MMP3;MMP13;CXCL2;TNFRSF12A;LCN2;PTX3;HP;SERPINE1;CX3CL1;SELP;FGF9;TFPI2;BDKRB1;NOS2;TLR2;MMP9;HSPA1A;CXCL3;IL6
pregnancy	6.51E-12	GJA5;PTGES;CXCL2;PCP4;TNFRSF9;PTX3;ATF3;HP;RGS2;PLA2G2A;STAR;MMP9;MMP3;ABCG1;TFRC;RND1;LCN2;NR4A1;ADH1C;SERPINE1;CFLAR;SELP;CX3CL1;TFPI2;NOS2;TLR2;HSPA1A;IL6
apoptosis	6.75E-12	GJA5;CYP7B1;CXCL2;TNFRSF9;SLC4A11;TNFRSF12A;PTX3;EGLN3;HP;RGS2;SDCBP2;MAPK8IP3;PLA2G2A;STAR;STEAP4;MMP3;CYP26B1;SH3KBP1;TGM1;DUSP5;LCN2;PDE1A;CFLAR;CX3CL1;C8orf4;NOS2;TLR2;HSPA1A;CXCL3;MMP13;PTGES;PCP4;PPM1E;NFKBIZ;NR4A3;ATF3;SLC16A3;NUP210;FANCD2;BTG2;CD180;MMP9;BAALC;ZC3H12A;ABCG1;TFRC;EVA1A;BANP;NR4A1;PRG4;SERPINE1;SELP;DDIT3;C1QTNF3;FGF9;TFPI2;BDKRB1;GALNT14;RNASE1;FCER1G;C3;IL6;RASD1
neutrophil migration	7.09E-12	MMP3;GJA5;CXCL2;TNFRSF12A;LCN2;PTX3;SERPINE1;SELP;CX3CL1;BDKRB1;NOS2;TLR2;C3;STEAP4;MMP9;CXCL3;IL6
cell survival	1.44E-11	MMP13;CXCL2;CYP7B1;PTGES;TNFRSF9;SLC4A11;NFKBIZ;TNFRSF12A;ATF3;NR4A3;HP;EGLN3;SDCBP2;FANCD2;SH3BP4;BTG2;MMP9;ZC3H12A;MMP3;ABCG1;CYP26B1;TFRC;SH3KBP1;SLC5A3;TGM1;LCN2;DUSP5;MLH3;NR4A1;SERPINE1;CFLAR;CX3CL1;FGF9;DDIT3;C1QTNF3;TFPI2;FCER1G;NOS2;TLR2;C3;HSPA1A;RASD1;IL6
**cell death**	**1.71E-11**	**GJA5;CXCL2;SLC4A11;TNFRSF9;TNFRSF12A;PTX3;EGLN3;HP;RGS2;SDCBP2;PLA2G2A;STEAP4;MMP3;SH3KBP1;TGM1;LCN2;CFLAR;CX3CL1;C8orf4;NOS2;TLR2;HSPA1A;PTGES;PCP4;PPM1E;NFKBIZ;NR4A3;ATF3;NUP210;FANCD2;BTG2;CD180;MMP9;ZC3H12A;ABCG1;TFRC;EVA1A;BANP;NR4A1;SERPINE1;FGF9;DDIT3;SELP;TFPI2;BDKRB1;RNASE1;FCER1G;C3;IL6**
blood vessel permeability	3.01E-11	MMP3;LAMC2;PTGES;CXCL2;LCN2;TNFRSF12A;PTX3;HP;NR4A1;SERPINE1;SELP;BDKRB1;NOS2;TLR2;C3;MMP9;HSPA1A;IL6
neutrophil extravasation	4.25E-11	SELP;BDKRB1;CXCL2;LCN2;PTX3;C3;MMP9;CXCL3;IL6
neuroprotection	4.94E-11	MMP3;MMP13;CXCL2;PCP4;ATF3;HP;NR4A1;RGS2;SERPINE1;CFLAR;FGF9;CX3CL1;DDIT3;BDKRB1;NOS2;STAR;BTG2;MMP9;HSPA1A;IL6;ZC3H12A
apoptosis of neutrophils	8.44E-11	CXCL2;TNFRSF9;LCN2;EGLN3;NR4A1;SERPINE1;DDIT3;NOS2;TLR2;MMP9;HSPA1A;IL6
monocyte adhesion	9.40E-11	ABCG1;GJA5;CXCL2;TNFRSF9;NR4A3;NR4A1;SERPINE1;SELP;CX3CL1;TLR2;C3;HSPA1A;CXCL3;ZC3H12A;IL6

Table S3 KEGG pathway significantly related the 29 DEG potentially regulated by JNK/p38 and related to cell death

KEGG pathway	p value	genes invovled in the KEGG pathway
Chagas disease	1.76E-07	SERPINE1,TLR2,NOS2,IL6,CFLAR
**Cytokine-cytokine receptor interaction**	**9.32E-06**	**TNFRSF12A,TNFRSF9,CXCL2,IL6,CX3CL1**
Toxoplasmosis	1.32E-05	TLR2,NOS2,HSPA1A,PLA2G2A
Malaria	2.92E-05	TLR2,SELP,IL6
MAPK signaling pathway	1.00E-04	HSPA1A,NR4A1,DDIT3,PLA2G2A
Rheumatoid arthritis	1.00E-04	TLR2,MMP3,IL6
Pathways in cancer	2.00E-04	MMP9,EGLN3,NOS2,IL6
Amoebiasis	2.00E-04	TLR2,NOS2,IL6
Prion diseases	7.00E-04	IL6,HSPA1A

Table S4 Transcription factors potentially regulating the 91 genes dysregulated by cyclic mechanical stretch in RASMC

Transcription factor	Track id	NES	Target genes
STAT1	wgEncodeSydhTfbsHelas3Stat1Ifng30StdPk.narrowPeak.gz	4.69	C8orf4,CXCL2,CX3CL1,C3,ABCG1,STEAP4,PLA2G2A,SDCBP2,GJA5,NOS2,DUSP5
MEF2A	wgEncodeHaibTfbsSknshMef2aV0422111PkRep1.broadPeak.gz	4.47	SERPINE1,NR4A3,RASL11B,ATF3,TNFRSF12A,TLR2,NR4A1
